# Translation of Polymeric Microneedles for Treatment of Human Diseases: Recent Trends, Progress, and Challenges

**DOI:** 10.3390/pharmaceutics13081132

**Published:** 2021-07-24

**Authors:** Prateek Ranjan Yadav, Monika Nasrin Munni, Lauryn Campbell, Golam Mostofa, Lewis Dobson, Morayo Shittu, Sudip Kumar Pattanayek, Md. Jasim Uddin, Diganta Bhusan Das

**Affiliations:** 1 Department of Chemical Engineering, Loughborough University, Loughborough LE11 3TU, UK; Prateek.Ranjan.Yadav@chemical.iitd.ac.in (P.R.Y.); lauryncampbell99@outlook.com (L.C.); l.j.dobson-17@student.lboro.ac.uk (L.D.); morayoshittu@hotmail.co.uk (M.S.); 2 Chemical Engineering Department, Indian Institute of Technology, Delhi 110016, India; sudip.kumar.pattanayek@chemical.iitd.ac.in; 3 Drug Delivery & Therapeutics Lab, Dhaka 1212, Bangladesh; monikamunni80@gmail.com (M.N.M.); mostofa.2471@gmail.com (G.M.); 4 Department of Pharmacy, Brac University, 66 Mohakhali, Dhaka 1212, Bangladesh

**Keywords:** human disease, polymeric microneedle, transdermal drug delivery, vaccine delivery

## Abstract

The ongoing search for biodegradable and biocompatible microneedles (MNs) that are strong enough to penetrate skin barriers, easy to prepare, and can be translated for clinical use continues. As such, this review paper is focused upon discussing the key points (e.g., choice polymeric MNs) for the translation of MNs from laboratory to clinical practice. The review reveals that polymers are most appropriately used for dissolvable and swellable MNs due to their wide range of tunable properties and that natural polymers are an ideal material choice as they structurally mimic native cellular environments. It has also been concluded that natural and synthetic polymer combinations are useful as polymers usually lack mechanical strength, stability, or other desired properties for the fabrication and insertion of MNs. This review evaluates fabrication methods and materials choice, disease and health conditions, clinical challenges, and the future of MNs in public healthcare services, focusing on literature from the last decade.

## 1. Introduction

Following the approval of the first transdermal patch for scopolamine administration by the FDA in 1979, as an alternative to oral administration of medications and hypodermic injections, the distribution of transdermal pharmaceuticals has gained considerable interest [1]. This is due to the numerous benefits, including improved dose reliability and control, enhanced patient engagement, and reduced clinical side effects [2]. Currently, there are numerous transdermal gels, ointments, and patches that can be applied to the skin [3]. The anatomical site chosen for these applications depends on the drug used and the rate of drug diffusion at the site of application [4]. For example, when the Alza Corporation introduced the first testosterone patch for men with hypogonadism, it was designed specifically to be worn on the scrotal tissue for its high permeability [5], while fentanyl patches were first designed for application on the upper thigh for pain management [6].

The transdermal drug delivery (TDD) systems such as the above have a range of constraints in their applications [3]. In particular, they are unable to deliver certain drugs across the skin at the desired therapeutic rates because of the poor permeability of the *stratum corneum* (SC) to these drugs [7,8]. The skin barrier only allows lipophilic and low molecular weight compounds (<600 Da) to pass through [9]. Therefore, many attempts at improving drug permeability by physical and chemical approaches have been investigated previously. Chemically, the use of chemical penetration enhancers has been shown to improve lipophilicity and, therefore, the bioavailability of the drug. However, there are problems of skin irritation and loss of dose associated with them [10]. Alternative methods such as iontophoresis [11], sonophoresis [12], and electroporation [13] can physically disturb the SC of skin, reducing its resistance to drug permeability. However, these methods are costly and require a difficult set of guidelines for effective drug delivery, which make them less user-friendly. To this end, microneedles (MNs) promise to be a more cost-effective and patient-friendly TDD system for the delivery of a host of drugs. MNs can pierce the SC and create transient microchannels that actively disperse foreign molecules through the blood [14]. Without disturbing the nerves of the underlying dermis and destroying blood vessels, MNs may also be optimized to enter the specified depths in the skin [15]. Thus, MN therapy allows for a minimally invasive molecule delivery into the skin, unlike the traditional methods for transdermal delivery of pharmaceuticals [16].

MNs can be produced from a range of materials such as metals, silicon, carbohydrates, and polymers [17,18,19,20]. Microneedles fabricated from biodegradable and biocompatible materials such as polymers present major benefits i.e., low cost, non-toxicity, and a range of physicochemical and mechanical properties. The biodegradable and biocompatible nature of a polymer-based MN (also called polymeric microneedles) is important as the MNs penetrate biological barriers and are exposed to bodily fluids and tissue. In this context, the nontoxic nature of MNs should comply with the United Nations’ Sustainable Development Goal 3—good health and well-being [21].

**Figure 1:**
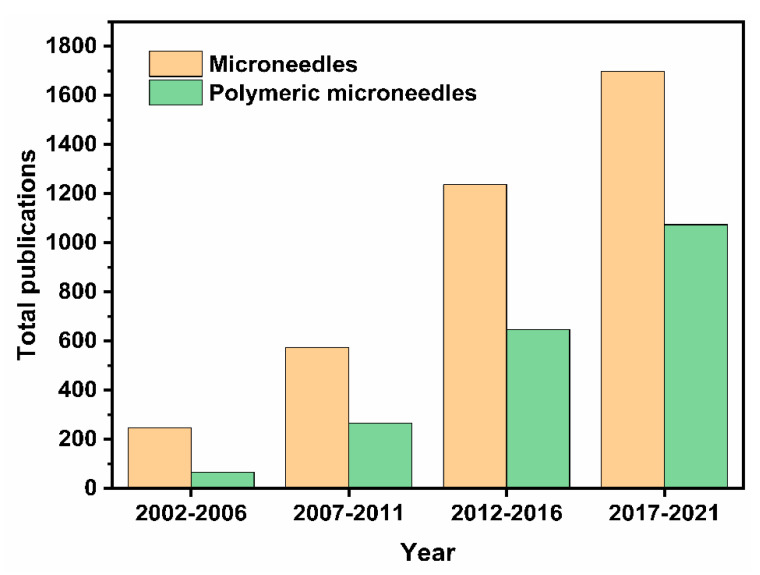
Bar charts displaying the total number of publications on MNs, and the number of MN publications related to polymeric MNs over the 5-year intervals. Information accessed on 26 June 2021 at www.scopus.com.

In the last two decades, MNs have become an increasingly popular study topic. This was evidenced in our analysis, which demonstrated that the number of publications obtained using the database Scopus with the search term ‘‘microneedle’’ has increased steadily. Figure 1 shows the number of these publications across different time periods in the last 20 years. This suggests a progression, and it can only be assumed that MN will continue as a technology in the future. The advanced search with term “microneedle” and “polymer” gave us the total number of publications that are related to polymeric MNs for that period. Figure 1 shows that the interest in polymeric MNs within the realm of MN drug delivery is increasing. For example, during 2017–2021, ~65% of the publications related to polymeric MNs. With the amount of research into TDD in conjunction with polymeric MN use continually growing, the potential of polymeric MNs is expected to increase.

However, several challenges must be addressed for these MNs to find widespread medical applications. Challenges include skin irritation, microbial contamination, lack of mechanical strength of biomaterials, the quantity of drug loading, and the delivery of macromolecules with high hydrophilicity [22]. There is also a need to form an optimized compromise between painlessness and penetration [23]. Thus, without the formation of accurate mathematical models for the release of specific drugs, the viability in the performances of different MNs for treating different diseases is difficult to ascertain. Researchers have now performed modeling and simulation studies on polymeric MNs to optimize drug delivery [24]. At the same time, it has been argued that modeling has the potential to reduce the financial and time cost of MN manufacturing [21].

With the ongoing MNs’ progress toward commercialization, there is a greater need to address the issues surrounding their translation from the laboratory to the end-user. In addressing this point, this review aims to discuss the latest trends, progresses, and challenges of polymeric MNs specifically, e.g., key marketed products, drug delivery mechanisms, polymer kinetics, fabrication techniques, materials used and classifications, MNs evaluation techniques, preclinical and clinical trials, and others. Various drugs administered using MNs for different diseases and ailments along with MN compositions are also discussed briefly to motivate the review topics further. At the end of the review, we conclude that for a successful translation of MNs, one needs to consider all these aspects successfully.

## 2. Polymeric Microneedles

**Figure 2:**
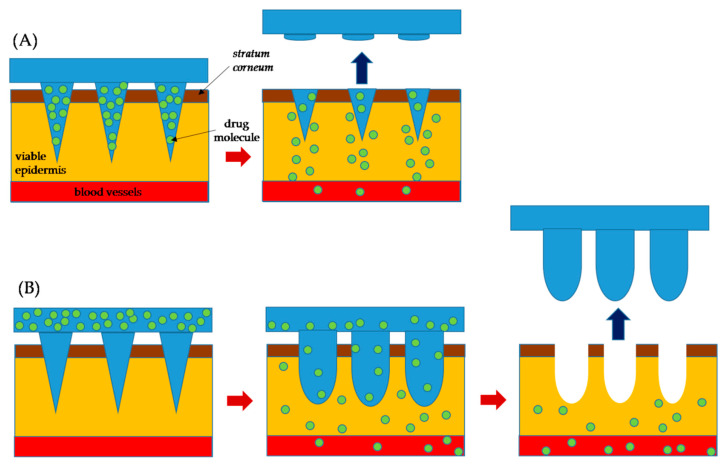
A schematic diagram for the mechanism of drug delivery: (**A**) dissolving MNs and (**B**) swellable MNs. The MN completely dissolve in skin in the case of (**A**), while there is no polymer dissolution in skin for the case of (**B**).

Polymeric MNs have been shown to be a strong technology to provide chemical molecules with clinical effectiveness and large complex biotherapeutic systems [25]. The polymeric MNs have also opened up a whole new horizon by offering many advantageous features as contrasted with silicone, metal, and other MNs [26]. They possess both the biodegradable and biocompatible properties and other benefits such as low cost, a wide selection of physicochemical and mechanical properties, and decreased risk of material build-up in the skin [27]. Polymeric MNs can be further separated into two categories of dissolving MNs and swellable microneedles. In the case of dissolving MNs, the drug is loaded inside the microneedle domain. Dissolving MNs absorb the skin water and completely dissolve in the skin which results in the release of the drug from the needle into the skin (Figure 2A). Swellable MNs have a reservoir attached at the base which contains drug in a lyophilized form (Figure 2B). Swellable MNs absorb local moisture within the skin, opening the polymeric matrix, and allowing the drug to diffuse from the reservoir into the skin. The swelled polymer matrix remains attached to the MN base throughout the delivery process, in which there is no polymer dissolution in the skin. We use the words “swellable” and “hydrogel forming” interchangeably as most of the swellable MNs are prepared using hydrogels. As the needle arrays either dissolve or soften, disposal of the medical waste can be executed safely without risk of stick injuries or contamination. Polymeric MNs are ideal for preserving the bioactivity of thermally unstable drugs such as protein vaccines and enzymes while at the same time minimizing the associated costs for cold storage [28]. There is a wide range of polymers with different swelling, degradation properties, and responsiveness to physical and biological stimuli. MNs made from these polymers allow control of the physicochemical properties and pharmacokinetics of drug molecules and performance in the skin for various biomedical applications [29].

## 3. Material Choice and Drug Release Kinetics of Polymeric Microneedles

The choice of material for MN preparation and drug release kinetics has key roles in translating polymeric MNs to commercial applications for the treatment of human diseases. Becton Dickinson (BD), United States of America (USA), Georgia Institute of Technology, USA, and Alza Company, USA, led studies on the development of MNs for medication administration in the 1990s. Metal or silicone was initially used as the raw material to produce MNs [30]. Nevertheless, researchers began using other materials that were biologically viable, lighter, and more renewable [31]. Biodegradable polymer MNs were developed in 2003 for the biodegradability method used in TDDs [32]. A list of the polymers for MN with competitive features is included in Table 1.

Due to its cost, consistency, biodegradability, hygienic application, swelling, and dissolving capability, polymers are favored [33]. Polymers are primarily used in dissolving and swellable MN arrays [34]. The in vivo breakdown creates nontoxic subproducts in biodegradable MNs. This decreases the chance of infection in the body [35]. The polymeric MNs can be classified based on compositions, construction, in vivo efficiency, and ingredients [36]. The use of biodegradable polymers is important for the management and analysis of the continuous release profile of the products in terms of the rates of decline [37]. Common components that are widely used in dissolving polymeric MNs included sodium hyaluronate, which is naturally found in the skin, sodium carboxy-methynyl cellular, poly(vinylpyrrolidone) (PVP), carboxymethyl cellulose (CMC), hydroxypropyl methylcellulose (HPMC), sodium alginate, as well as other components [38,39]. Furthermore, bio-responsive polymers such as hyaluronic acid (HA), polyvinyl alcohol (PVA), and alginate, cross-linking methacrylate are also used [40]. Biocompatible, non-immunogenic, mechanically complex materials should be perfect for polymeric MN materials [41]. A list of the polymers for MNs with competitive advantages are included in Table 1 and Table 2.

Each polymer used within an MN is characterized by its potential for strength, penetration, and continuous release of drugs [42]. MN penetration through the skin is the greatest obstacle for polymer MNs [43]. In contrast to non-dissolving materials such as silicon or copper, the mechanical strength of water-soluble polymers is often lower, and drug encapsulation may further weaken the strength of MNs [44]. Flash modulus and polymer MN fractures are important for mechanical resistance, as the insertion capability of the polymer-based MN is reflected here [27]. Researches can also combine the mechanical power of two or more polymers and additional materials [45]. Furthermore, during polymer selection, the target tissue for the MN must be called transdermal or non-transdermal [46,47]. Aimed at soft tissues that cannot withstand pressure from the high-strength MN implant, careful balancing of strength and versatility must be considered [48]. Environmental moisture is also an important consideration, as greater moisture levels decrease the resistance of the MNs by the polymer and humidity [49].

Moreover, the active ingredient loaded into the polymeric MNs may improve mechanical strength [50]. The mechanical strength of the MNs can be decreased if the drug dispersed in the base plate of the MN arrays, which were shown by cracking the base plate after mechanical assessment, is placed in the MN not only to overcome the mechanical strength problem but also to decrease drug wastage [51]. The substance used for producing MNs is the most important design element, as it determines the strength of the mechanism and the release effect of MN drugs [52]. The substrate density, MN height, diameter width, and base of the MN are other factors to be considered [43]. However, manufacturing is constrained by the time-consuming steps typically needed, for example, master preparation, mold creation, and plasticization of thermoplastic polymers above their transition temperature. Therefore, thermal-free products cannot be used, and thus polymer MNs are usually manufactured with the aid of shaping techniques [53]. Researchers tackle the problem of thermal-friendly medicines by pouring the medication and polymer solutions into the mold under vacuum or heat [54]. In Table 1 it has been shown that the favorable materials and their benefits after using them to develop MN. It has been shown that the challenges that must be overcome for developing a good quality MN.

**Table 1:** General properties of typical polymers used in the preparation of MNs.

Material *	Benefits	Limitations	Fabrication Technique	Reference
PVA	Low material cost Different grades and molecular weight (MW) available Composites provide good plasticity and dissolvability Nontoxic Can be used to prepare dissolving as well as swellable MNs	Cross-linkers used such as glutaraldehyde or formaldehyde may reduce biocompatibility Absorbs water quickly	Molding Fused deposition modeling (FDM)	[55,56]
PLGA	Offer excellent applicability Used in preparing dissolving MNs	High material cost	Molding Micro milling Hot embossing	[57,58]
HA	Offers rapid dissolution Used in preparing dissolving MNs as well as glucose-responsive MNs	Can cause skin irritation	Micro molding	[59]
Gantrez^®^	Copolymer of methyl vinyl ether and maleic anhydride Excellent swellable capacity Mostly used for preparing swellable MNs	High material cost	Micro molding	[36]
PCL	Exceptional thermal stability Easily processed High permeability	Slow degradation	Micro molding Hot embossing 3D printing	[60,61]
PEGDA	Easily customized to include biological molecules	High material cost	Photolithography	[62]
PGA	Fast degradation High tensile strength Excellent fiber-forming ability Excellent mechanical properties	High material cost	Injection molding Lithography Fused deposition modeling (FDM)	[61,63,64]
PLA	Easily processed Adjustable degradation rates Excellent physical and mechanical properties Mainly used in solid and coated MN fabrication	High material cost Slow degradation	Molding, FDM	[65,66,67]
PVP	Different grades and MW available Composites provide good plasticity and dissolvability Low cost	Difficulty scaling up the process efficiently	Molding Photopolymerization	[63,68]

* Reference: HA: hyaluronic acid; PEEK: polyether ether ketone; PEG: polyethylene glycol; PEGDA: poly (ethylene glycol) diacrylate; PGA: polyglycolide; PLGA: poly (lactic-*co*-glycolic acid); PMVE/MA: polymethyl vinyl ether-alt-maleic anhydride; PVA: polyvinyl alcohol; PVP: polyvinylpyrrolidone.

**Table 2:** Various polymers and fabrication techniques for dissolving and swellable MNs.

Type of Polymeric MN	MN Construction Polymers	Fabrication Techniques
Dissolving MNs	Chitosan [69]; Carboxymethyl cellulose, CMC [70]; Dextran [71]; Dextrin [72,73]; Hyaluronan, HA [74]; Polyvinyl alcohol, PVA [34] Polyvinylpyrrolidone, PVP [75] Sugars [76]	Micro molding [54]. Drawing lithography [70]; Continuous liquid interface production, CLIP [77]; 3D Printing [2,78]; Injection molding [79]; Hot embossing [80,81]; Photolithography and etching [82];
Swelling MNs	Hyaluronan, HA [83]; (Hydroxyethyl) methacrylate, HEMA [84]; Polyvinyl alcohol, PVA [55]; Poly(styrene)-block-poly (acrylic acid), PS-b-PAA [85]	Crosslinking via: Lyophilization [55]; Heating [84]; UV exposure [86];

### Polymer Dissolution Kinetics

Kinetics can be defined as the study of the rate of change of concentration. Dissolution is described as the process of a substance moving from a solid state to forming an aqueous solution. Hence, dissolution kinetics is the study of the rate of change of concentration due to a substance dissolving [87]. Therefore, research into dissolution kinetics is a vital part of modeling dissolving MNs. The dissolution of the polymer that forms the dissolving MNs can be prompted by several stimuli [88]. In this section, we briefly discuss the key parameters, namely, temperature, ultraviolet, pH, and moisture responsive polymers that affect the performances of dissolving MNs.

Photothermal Responsive. Photothermal responsive polymers are polymers that melt in the presence of photons of a specific range of wavelengths due to the absorption converting light energy into thermal energy [87]. Chen et al. [60] produced polycaprolactone MNs with silica-coated lanthanum hexaboride (LaB6@SiO_2_) nanostructures incorporated. The purpose of the LaB6@SiO_2_ was to absorb near-infrared ray (NIR) wavelengths. Therefore, when exposed to NIR, the structure heated, causing the MN to melt at 50 °C. This approach allows for the release of active pharmaceutical ingredients upon demand using externally applied NIR [89].

pH-Responsive. pH-responsive polymers are polymers that allow for the transfer of a substance as a direct result of acidic or alkaline conditions. Ke et al. [90] developed hollow MN arrays containing microspheres, which encapsulated two model drugs and sodium bicarbonate. The microspheres had a thin poly(d,l-lactic-*co*-glycolic acid) (PLGA) external membrane, which protons could diffuse through. Thus, when the hollow MN array delivered the microspheres into the skin, which is naturally acidic, the protons reacted with the sodium bicarbonate within the microspheres. This formed CO_2_, which generated pressure within the microsphere. The pressure increased until the microsphere membrane ruptured, releasing the encapsulated drugs [90]. Ullah et al. [91] implemented the foundations of the microsphere technology into a polymeric MN array. Ullah et al. [91] created a MN array containing pores of sodium bicarbonate mixed with the drug. The MN array had a thin PLGA external membrane that protons could diffuse through. Therefore, when introduced to the acidic environment of the skin, the pressure was generated inside the pore. When the pressure was large enough, the membrane ruptured, releasing the encapsulated drug [91].

Moisture Responsive. Moisture responsive polymers are polymers that undergo dissolution due to hydrolysis. Moisture responsive dissolving MNs can either be designed for an instant release or a controlled release system [92]. Commonly, water-soluble polysaccharides are used as the construction polymer for instant release systems. Examples used in instant release systems include polyvinyl alcohol (PVA), polyvinylpyrrolidone (PVP), hyaluronan (HA), dextran, and carboxymethyl cellulose (CMC). However, polysaccharides are also used in dissolving MNs for the controlled release of a drug due to their biocompatibility and stability [87]. Koh et al. [93] developed a method of delivering basic mRNA using a polyvinylpyrrolidone dissolving MN patch (RNA patch). The RNA patch was shown to completely preserve the physical and functional integrity of the encapsulated mRNA for at least two weeks. The kinetics of the RNA patch were comparable with subcutaneous injections and could be increased with longer MN lengths. The use of moisture responsive polyvinylpyrrolidone in a dissolving MN array showed potential for the safe and efficient delivery of mRNA-based therapeutics [93].

## 4. Modeling and Optimization for Designing Polymeric MNs

MNs are ultra-small and minimally invasive drug-delivery devices requiring regulated, precise, and repeatable injections into human skin [1]. During injection into the skin of the hard SC layer, complex injection methods are pursued because of the high flexibility and elasticity of the skin [94]. Lutton et al. [95] proposed three basic requirements in considering universal acceptance criteria for MNs: (1) they must pierce the skin, (2) they must penetrate and remain in the skin to dissolve or interact in the skin while delivering the therapeutic agent, and (3) they must act within the specified timeframe and must be able to dissolve or else be removed [95].

**Figure 3:**
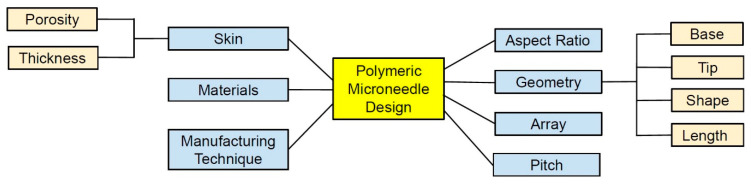
Several parameters affecting the design and performance of polymeric MNs.

There are different parameters which shape the design and effectiveness of MN devices, as shown in Figure 3. Therefore, to successfully insert MN devices with various materials, geometric characteristics, and array size, the effect of MN design on the skin needed to be established [14]. The selection of materials capable of controlling the release and drug stability during manufacturing are central to the design strategy are the key for safe and efficacious MNs [66,96,97]. The materials (e.g., silicon, polymer, metals, or carbohydrates), arrangement on the substrate (e.g., radial, triangular, square, or hexagonal), and the geometry (i.e., base diameter, tip diameter, base-to-tip ratio, and center-to-center spacing) of the MNs can affect the penetration depth [36,82]. Controlling the length, sharpness, arrangement, and puncture rate of the MNs is important to meet the criteria set by the FDA for an MN system as a medical device [98]. Geometries of the MN, such as shapes, aspect ratios, and tip radius, affect the skin insertion capability of polymeric MNs [99]. For example, pyramidal shapes exhibited better mechanical strength than those with conical shapes due to their larger cross-sectional area with the same base width [2,100]. Also, the mechanical strength of pyramidal-shaped MNs can be further improved by increasing the base width and decreasing the aspect ratio [40]. Shorting the tip length and widening the base width increased the failure force [101]. Chen et al. [102] showed that the smallest aspect ratio exhibited the highest mechanical strength and the deepest insertion depth in the pyramidal MNs. The mechanical strength for MNs with the same aspect ratio but different dimensions does not vary significantly [102]. These results support that both shapes and aspect ratios are crucial variables for the mechanical properties of MN. It should be noted that widening the bases for decreasing the aspect ratio would increase the difficulties in achieving efficient skin insertion [40]. For better penetration, increasing the sharpness of the tip increases the penetration depth considering sufficient mechanical strength is present [103,104]. Insertion force for MN decreases by increasing the insertion speed, whereas increasing center-to-center interspacing of MN tips decreases the insertion force [104,105]. Overall, polymeric MNs with a pyramidal shape, smaller aspect ratio, and sharper tips exhibit good skin insertion [40].

### 4.1. Role of Skin on MN Insertion Behavior and Drug Delivery

**Figure 4:**
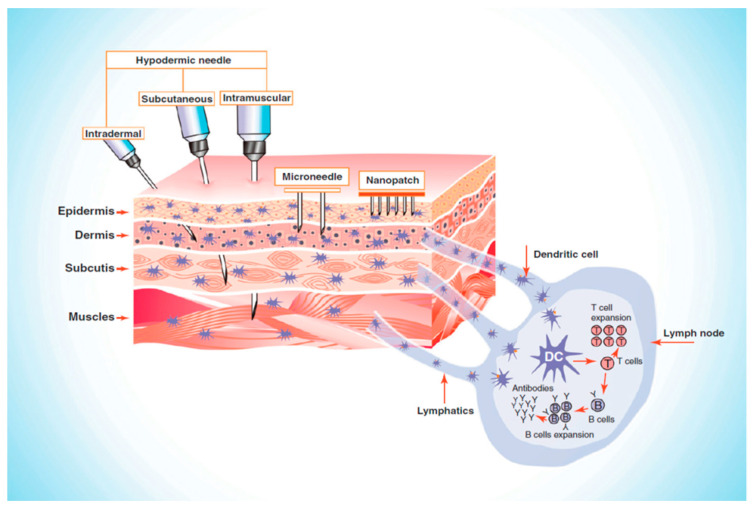
Illustrating the comparative penetration of hypodermic needle and microneedles (MNs), Reproduced with permission from [113], Elsevier, 2011.

Numerous factors, including age, gender, ethnicity, anatomical area, and hormonal balance affect the thickness of the skin [106]. MN patches pierce the SC, thus bypassing the barrier layer and delivering 100% of the loaded drug without pain [107]. Then, the drug is directly delivered into the upper dermis layer, which distributes into the systemic circulation, and on reaching the site of action, produces a pharmacological response [9,108,109]. Most MNs are 150–1500 μm long and 50–250 μm wide, with 1–25 μm tip thickness. The MN length of up to 1500 μm is sufficient to release the drug into the epidermis since the epidermis is up to 1500 μm thick [110]. MNs larger than 1500 μm in length can go deep into the dermis, damage the nerves, and therefore cause pain [110,111]. Laurent et al. [112] found that regardless of gender (205 women and 137 men), age (18–70 years), and ethnicity (Asian, Caucasian, and African American), the mean skin thickness of the suprascapular, deltoid, and waist ranges from 1.55 to 2.54 mm and the suitable MN length for intradermal vaccine delivery is 1.5 mm [112]. As presented in Figure 4 [113], MNs only penetrate the epidermis to deliver the drug where hypodermic needles need to reach up to the muscle level for drug delivery.

Longer MNs can penetrate deeper and create longer micro-holes in the skin. As a result, more drugs can effectively move across the *stratum corneum* (SC), increasing drug permeability through the pathways created by the MNs [36,48,114]. Increasing the MNs height can promote the vertical and hardly affect the horizontal drug diffusion. The dosage of drug administered can be controlled by adjusting the height of MNs for skin pretreatment, and thus more drug distribution occurs in the skin [24,48]. Higher density MN patches can induce higher drug diffusion horizontally as higher densities of the MNs create more micro-holes and distribute more drugs through the micro-holes into the skin. Therefore, increasing MN density is an effective way to increase drug penetration for large scales of skin pretreatment with MNs [115,116].

To visualize and evaluate intradermal penetration depth, there are techniques such as histological sectioning, confocal microscopy, fluorescence microscopy, optical coherence tomography (OCT), X-ray computed tomography (micro-CT), and high-speed X-ray imaging [7,117,118,119,120,121].

### 4.2. Mathematical Modeling of Polymeric MNs

The mathematical models, in general, have been used mostly to verify the performance of the designed MNs [122]. The main objectives of mathematical modeling are to obtain the required design parameters to optimize the MN [123,124]. The establishment of a mathematical model that simulates the physical mechanisms occurring during transdermal drug delivery is essential for the medical industry as the development of such a model will enable key parameters that are difficult to measure be quantified. The most notable parameters include the rate of swelling and dissolution, insertion forces, drug release profiles, and MN efficiency (the percentage of drug released to drug initially encapsulated) [24,125,126,127,128].

When focusing on the skin, the major barrier-to-mass transfer within the skin is the SC. However, when modeling the MN-skin system, the full epidermis should be considered [129]. Andrews et al. [130] provided evidence for this statement as they showed the removal of the SC significantly increased the permeability of the drug. Also, the removal of the full epidermis increased drug permeability by another one to two orders of magnitude. The base membrane and tight junctions should also be considered when modeling as they may also provide resistance to mass transfer [130]. For an effective diffusion study of therapeutics using MN, the skin-related parameters (e.g., porosity, thickness, Young’s modulus, etc.) are needed to be included [127].

When modeling polymeric MNs, there are several factors associated solely with the MN that needs to be considered. The main consideration for the MN is the hydrolysis reaction occurring between the steric bonds of the polymer. The reaction results in a reduction of the molecular weight and describe the degradation of the MN array [21]. The geometry of the MN also needs to be considered [82]. However, as Sandrakov et al. [131] proved that conical MNs are the most optimal, it is logical to start with conical geometry as a standard when modeling. The length of the MNs, both tip and base diameter of the MNs, center-to-center spacing between two MNs, numbers of MNs in the array, and the distribution of the MNs in an array (square, diamond, triangle, rectangle, or exceptional design), etc. are responsible factors for MN performance [24,128]. The insertion force is further related to the MN tip angle and radius of the tip’s curvature [24,126]. Various mechanical parameters of MN, including Poisson’s ratio, Young’s modulus, ultimate tensile strength, etc. are used for evaluating insertion studies of MNs [132].

The other key component of modeling is the selection of physics that allows for the evaluation of drug-release profiles. The governing equation for the mass transfer of the drug is Fick’s law [12]. Once the physics has been selected and the parameters have all been defined, the model can be simulated on computational software. The most common software packages used are MATLAB^®^ and COMSOL Multiphysics^®^ [133,134]. The water absorption rate should also be considered while modeling the dissolving and swellable MNs [135]. Gomaa et al. [122] demonstrated that molecular weight is one of the significant factors that affect the molecular diffusion rate in the skin in general, and MN pierced skin.

Zhang et al. [136] developed a mathematical model to quantitatively predict the transient behavior of the drug delivery using solid polymeric MNs. To achieve this, Zhang et al. [136] applied mixture theory where the skin (biological tissue) was treated as a multi-phase fluid-saturated porous medium, using conservation equations to characterize the mathematical behavior of the tissue. The model [136] also included drug absorption by blood capillaries and tissue cells, modeled as a moving interface along the flowing pathway. The mathematical model was used to help solve problems with drug absorption within the blood capillaries and tissue cells. The Zhang et al. [136] model allowed for a greater understanding of the mechanics behind drug delivery from MNs. However, interest has moved away from solid MNs, and the focus is upon dissolving MNs.

Kim et al. [22] developed a mathematical model which predicts the quantity of drugs delivered via the dissolution of dissolving MNs. The initiative part of their model was the inclusion of a biological membrane. The model used governing equations and numerical solutions to estimate fentanyl concentrations within different layers of the skin and simulated the dissolution of the dissolving MNs. The model agreed with predictions created from dimensionless parameters. The predictions included the pitch of the dissolving MNs being inversely proportional to the fentanyl delivered and the insignificance of elimination kinetics to the dissolution of the dissolving MNs. The model also had an optimization algorithm applied to recover parameters that represented the experimentally obtained data most accurately. Kim et al. [22] concluded that the algorithm applied may provide a useful tool to characterize the drug delivery regimen by dissolving MNs.

**Figure 5:**
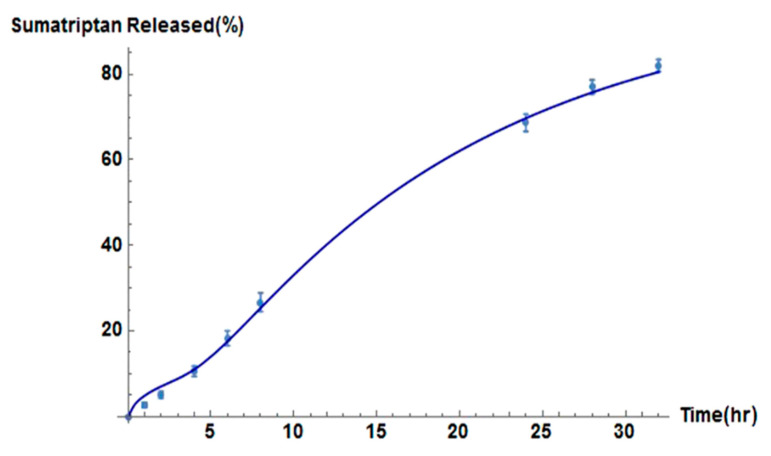
Relationship between sumatriptan released and time by the model (solid line) and in vitro (solid dots) for dissolving MN formulations, reproduced with permission from [32], Elsevier, 2020.

Recent research has focused on the advancement of dissolving MN technology to increase the therapeutic effects of drug molecules, which requires a further understanding of the transport mechanisms within the skin. Ronnander et al. [32] developed their model to assist researchers when evaluating the administration of sumatriptan with a limited dataset. The model simulates the dissolution of pyramidal-shaped dissolving MNs and the diffusion of sumatriptan using governing equations, as shown in Figure 5 [32]. The results obtained by Ronnander et al. [32], shown in Figure 5, suggest that the mathematical model developed agrees with the experimental data. Conclusions drawn by Ronnander et al. [32] included reducing pitch width significantly, which increased sumatriptan diffusion within the skin, coinciding with the findings of Kim et al. [22]. The PVP polymer concentrations also had a significant effect upon dissolution, and drug loading had a lesser effect upon dissolution [32].

**Figure 6:**
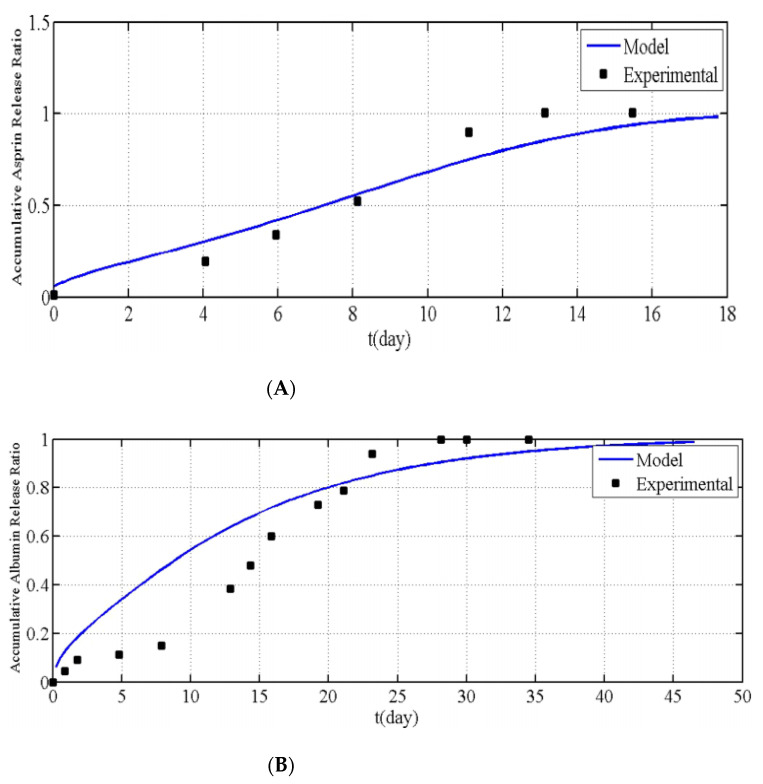
Relationship between the accumulative (**A**) aspirin and (**B**) albumin release ratio vs. time for modelling results and experiments, as reported by Chavoshi et al., reproduced with permission from [21], Springer Nature, 2019.

Chavoshi et al. [21] developed their mathematical model using the same methodology as Ronnander et al. [32]. However, Chavoshi et al. [21] considered autocatalytic effects on their polymeric dissolution to predict drug release profiles. The drug release profiles obtained for aspirin and albumin are shown in Figure 6. From Figure 6, it is apparent that the model produced by Chavoshi et al. [21] does not match the experimental values as accurately as the model developed by Ronnander et al. [32]. Chavoshi et al. [21] concluded that the differences between experimental and model values arose from errors within the estimation of some parameters of the model. The need for estimation is due to the difficulty in finding the parameters in the exact same utilized conditions.

## 5. Polymeric Microneedle Manufacturing Techniques

The drug is normally embedded inside the needle through polymeric MNs, but swellable MNs may also distribute the medication from an external reservoir [137]. The drug is subsequently combined with a solvent to create a drug-containing solution. The composition of a solvent with a selected polymer forms a solvent for a polymer substance [80]. MNs may be made using a wide range of methods using a polymer-drug solution [32]. The choice of the construction technique of the polymer and the MN is dependent on the specifications of the MN patches needed [8]. A list of the building polymers and processing methods used in both dissolving MNs and swellable MNs is given in Table 3, in Section 8. The advancement of science and technology has aided the development of more versatile MN fabrication techniques in the past few decades [88,138].

**Figure 7:**
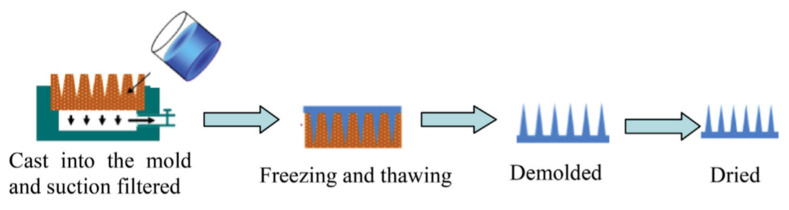
Polymeric MNs manufacturing using micro molding method, reproduced with permission from [150], Elsevier, 2016.

Micro molding is the preferred method for the preparation of polymeric MNs due to high reproducibility, convenience for scalable production, and cost-effectiveness [139]. This is because certain medications and vaccinations are thermolabile, and micro forming allows for the preparation of needles in mild conditions [51]. Usually, hot embossing methods, investment molding, and injection molding are used to fabricate degradable and insoluble MNs, but due to relatively high processing temperatures, the drug activity may be easily affected [140,141,142,143,144,145]. The casting method is currently the most used method to prepare dissolvable polymer MNs due to the advantages of low processing temperature, convenient fabrication process, and insignificant impact on drug activity [145,146]. Involved heat or UV irradiation in the micro molding method might reduce the activity of sensitive drugs [147]. Apart from the micro molding method, drawing lithography, droplet-born air blowing, electro-drawing, and 3D printing can achieve rapid (usually within 10 min) MN preparation as these do not require the use of a mold [70,77,148,149]. In Figure 7 [150], the basic steps for micro molding have been illustrated.

**Figure 8:**
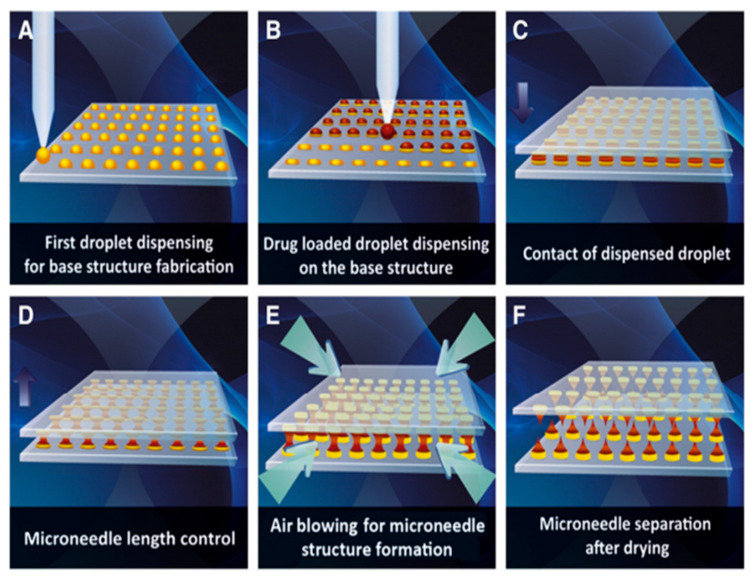
Schematic illustration of dissolving microneedle fabrication via droplet-born air blowing method. (**A**) Biopolymer dispensing on the flat surface for base structure fabrication. (**B**) Dispensing of drug-containing droplet over the base structure. (**C**) Contact of dispensed droplet by downward movement of upperplate. (**D**) Control of microneedle length. (**E**) Air blowing-mediated solidification of droplet to shape microneedle structure. (**F**) Separation of two plates producing dissolving microneedle arrays on upper and lower plates. Droplet-born air blowing (DAB) technology for fabricating polymeric MNs, reproduced with permission from [70], Elsevier, 2013.

Droplet-born air blowing (DAB) is a novel technique of fabrication where the droplet of a polymer is shaped into the MN through air blowing [70], as shown in Figure 8. The direct application of the air is for the solidification of the polymer droplet that helps in the formation of the shape of the MN [151]. This DAB technology allows the fabrication under conditions without the need for UV irradiation or high heat [152]. It requires a temperature of 4–25 °C and a brief period (≤10 min) [153]. The number of drugs can be controlled by regulating the pressure and time with the help of the droplet dispenser [154]. As a result, loading the drug in the MN is possible without drug loss, which facilitates the maintenance of the activity of the biological drugs [155].

Drawing lithography involves creating microstructures that are three-dimensional from two-dimensional (2D) materials [156]. The drawing lithography method can not only fabricate MNs with a high aspect ratio but also eliminates the need for molds and UV light irradiation, thereby avoiding the use of toxic photo-initiators in comparison with conventional micro molding [157]. However, in comparison to micro molding, the drawing lithography method exhibits much worse reproducibility [149]. Electro-drawing has emerged as an alternative fast and mild temperature strategy to the conventional use of stamp-based techniques for the fabrication of biodegradable polymer MNs [158].

**Figure 9:**
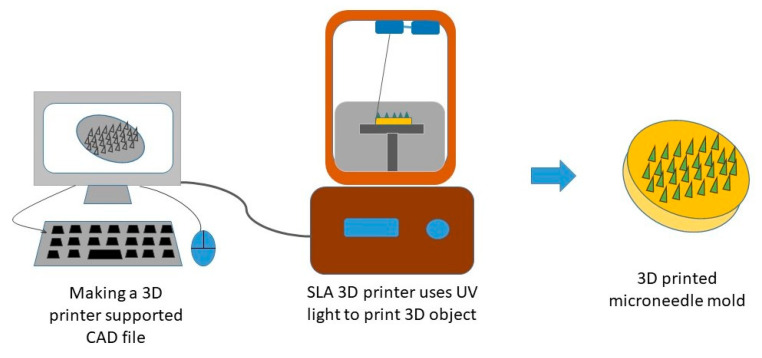
Illustration of the process involved in 3D printing of polymeric MNs. Adapted from [162], Springer Nature, 2019.

Most importantly, the mentioned conventional MN fabrication techniques can only fabricate MNs on a flat substrate surface; consequently, it is difficult to fabricate uneven or curved skin surfaces due to its decreased penetration efficiency and drug delivery amount [159]. The reason for the popularity of 3D printing technology has been attributed to the tunability and versatility that facilitates the personalized fabrication of physical models of the desired geometric shape with computer-aided design and computer-aided manufacturing [160,161,162]. This prototyping technology is based on layer-by-layer printing and superposition that provides high accuracy along with good reproducibility [163,164]. The most current and commonly utilized manufacturing technologies of 3D printing include inkjet printing, photopolymerization-based technique, and fused deposition modeling (FDM) for fabricating polymeric MNs [160,162,164,165,166,167]. Figure 9 [162] shows the steps for fabricating MN devices using 3D printing technology. Inkjet printing allows the selective deposition of droplets of the drug onto the MN surface with the help of thermal or piezoelectric-driven printing heads [168,169], and digital light processing (DLP), stereolithography (SLA), and two-photon polymerization (2PP) are the commonly used photo-polymerization-based techniques to fabricate polymeric MNs [170,171,172,173].

## 6. Characterization Techniques for Polymeric MNs

A drug can be loaded in suspension or dispersion form or encapsulated into MNs [174]. The medication may also be coated or sprayed as a patch with a silicone solution [175]. For a drug-loaded MN, various physicochemical characterizations, including the grade of particle size, Poly dispersion index, viscosity, and zeta potential may be measured according to the type of formulation used in MNs [176]. An MN patch is complete after preprocessing for the release of drugs, adhesion, and permeation checks [177]. Dynamic light dispersion, X-ray dispersion, and transmission electron microscopy technologies allow for the characterization of MN size, inner structure, and crystallinity [178]. Drug dispersion stability and MNs can be tested at a different temperature, pH, and physiological stimulation in vivo [179]. Additional testing is conducted on engineered MNs, such as solubility experiments, pharmaceuticals content, in vitro release, and biocompatibility analyses [180].

Morphological Evaluation. Scanning electron microscopic (SEM) studies of MN patches are the most common techniques to understand MN morphology [181,182]. Previous research has used SEM studies to examine MN size, shape, height, base, pitch, and other physical attributes [36,183]. For SEM analysis, coating the MNs with a gold solution is used for capturing a clear image using a low voltage (1.0 kV) to avoid any electrical charges on the MNs surface [2,184]. The images of coated MNs are captured digitally from a fixed working distance using different magnifications (e.g., 30, 80, 110, or 120×) [2,164]. A closer look at the MN patches reveals high-resolution architecture with fine and sharp tips [181,183,184,185,186].

Dimensional Evaluation. Different techniques have been used for the measurements of MN geometry and for calculating the MN tip radius, length, and height [187]. Optical or electrical microscopy is the most common technique [188]. An optical scanning microscope is used to visualize the array and tips of the MNs, which capture digital images to show an entire array of uniformly distributed MNs with the same height and sharp tips [181,182]. A better image of the MN is produced using 3D image analysis, which helps to control quality [137], and for this purpose, scanning electron microscopic (SEM) and laser microscope confocal microscopy have also been used [164,189]. The SEM creates a sample image by using an electron-focused beam that interacts with the atoms of the sample when scanning and provides information on the topography and structure of samples [190]. Conversely, a confocal laser microscope produces images that are high in resolution [191]. Confocal microscopy studies are performed for producing fluorescent imaging of the MNs aimed at characterizing the compartmental structure of MNs using different chromophores in the sample [181,182,184].

Mechanical Testing. An MN should be sharp and thin enough to reach the skin quickly and firmly enough not to rupture within the skin [192]. The force at which the MN loses its structural integrity and insertion force are two essential considerations for the secure and effective construction of the MNs [193,194]. The Instron 5848 Micro Tester has been used to determine the mechanical properties of the MNs [195]. For this purpose, MNs are placed directly on a loading cell for an axial fracture test, using an axial force [196]. A block of aluminum is used for the transverse fracture test [197] and with ethyl cyanoacrylate super-glue gel, the back layer of the MNs are then fastened [198]. For transverse forces on the MN, a metal sonde with a 1 cm blunt can be used. To measure bend, the MN array is contained above the loading cell, using two aluminum blocks [199] and the sample then adds strength to the middle of the backplate [200].

In vitro and In vivo Studies. For the in vitro study, diffusion cells have been used to determine drug permeation across the skin [201]. The skin of the porcine ear was used primarily in the experiment between the recipient and the donor compartment [202]. Here, a typical set of permeation profiles for MN-treated and untreated skin were compared [203]. For the in vivo study, hairless rats were used [166]. The effective technique used for anesthetizing the animal and then measuring the trans epidermal water loss (TEWL) before and after MN insertion is one of the key parameters to be considered [204].

Content Uniformity Studies. According to FDA guidelines, the drug contents of the MNs need to be evaluated accurately [98]. For example, content uniformity is critical for solid MN systems; however, achieving a uniform coating on MNs is challenging [106,138]. Coating uniformity, including the homogeneity of drugs and the smoothness of the coating on coated MNs, have previously been qualitatively evaluated using scanning electron microscopy (SEM) and polarized microscopy [205,206,207].

Stability Evaluation. The stability of MNs depends on the stability of the polymers and drugs individually [35,208]. To evaluate the storage stability of MN arrays, the MN arrays, or their base-plates (with no MN) were exposed to different relative humidity and store in a temperature-controlled environment for a specific time [56,209]. Durability, deformation, and suction prevention are important considerations for MNs, whereas particle size, crystallinity, and polymorphism need to be considered for drugs coated on or dissolved in MNs [208]. The stability of the vaccine is of immense importance because of the limitation for refrigeration [210,211,212,213]. Several MN systems contain a solid form of vaccines that can potentially overcome the stability issue [214,215]. The addition of a stabilizer can improve the thermostability of the vaccine, and the appropriateness of the stabilizer depends on the nature of the vaccine [216]. The trivalent subunit influenza vaccine in a dissolving MN patch, for example, was stable at 25 °C for more than 1 year and at 60 °C during freeze-thaw cycles and electron beam irradiation for more than 6 months [205].

Chemical Stability. Differential scanning calorimetry (DSC) has been used to observe the enthalpy-related changes in the constituent materials of MNs by increasing the temperature. The constituent materials were heated to 300 °C at a rate of 10 °C min^−1^ using DSC that was calibrated previously with standard materials [44,217]. Thermogravimetric analysis (TGA) was used to record the samples’ weight loss on increasing temperature. To evaluate the physical and chemical stability of the materials, the desired samples were heated from 25 °C to 350 °C at a rate of 10 °C min^−1^, and the weight loss was recorded during this time. Fourier transform infra-red (FTIR) spectroscopic analysis was used to record the vibrational changes of different functional groups present in the samples in following interactions with light over a specific wavelength range (400–4000 cm^−1^) [44].

## 7. Pharmacokinetic and Drug Release Behavior from Polymeric MNs

Polymeric MN technology has been successfully used to improve drug penetration through the skin layer [218]. This has been shown in in vitro skin models with increased absorption of larger molecules such as calcein [219]. Polymeric MNs encompass the functions of a transdermal patch and hypodermic needle, aiming to achieve their benefits and remove each of their drawbacks [29].

Animal Models. Most pharmacological studies are conducted through in vivo animal models [46,50,220,221,222,223]. The animal fur was removed from the anesthetized animal, and the MN patch was subsequently applied to the underlying skin. Next, the blood was collected for the detection of different biomarkers [50,223,224,225], and a punch biopsy was performed on the dorsal skin of the mouse post-treatment [226]. Hematoxylin-eosin stain can be performed to observe epidermal status, while improvements in the bundle of collagen and elastic fibers were prepared with trichrome stain of Masson and blue Victoria stain, respectively [227]. The immunohistochemical components were evaluated using UltraVision LP Large Volume Detector Systems HRP Polymer Package (Thermo Fisher Scientific, Waltham, MA, USA) to determine the regulatory effects of UV and micronodular RF applications on the collagen in the extracellular matrix [228]. However, the structure and pharmacological response in an animal model differs from humans [50,106]. The pharmacological and pharmacokinetic analyses aid in optimizing the design of MNs and drug delivery for better therapeutic effect [44,200,220,224].

Porcine Skin Cargo Delivery Tests. The amount of drug delivered from the MNs to the skin was assessed using Franz diffusion cells with abdominal porcine skin [44,153,200,223]. Collected Skin samples were then placed in a phosphate-buffered saline (PBS) (pH 7.4) system for 1 h, and the prepared MN patch is inserted into abdominal porcine skin (1000 μm thick) for 30 s to determine the diffusion rates and effectiveness into porcine skin [153,164,229]. Methylene blue was applied to the needles to test the delivery of cargo covered on polymeric MNs [230]. The pierced skin and MN patch were then mounted onto the donor compartment of a Franz diffusion cell and maintain a temperature of 37 °C [223]. To determine drug concentrations, sample fractions were taken at set intervals, and atomic absorption spectroscopy used for quantitative analysis of drugs [164].

Skin Recovery Process and Irritation. When an MN device is implanted and removed from the skin following treatment, it leaves behind micron-size pants [231]. The skin needs time to recover its barrier properties [232]. If the skin is sensitive, it can lead to mild to medium inflammation or allergies, redness, pain, swelling, and itching may cause discomfort for the patient [233]. Although the pores formed by microns in comparison to the hypodermic needle are small, microbial penetration is also less serious [153], and the pores will take time to rescreen [234]. These pores must be immediately resealed the active drugs, or contamination will occur [235]. An electrical impedance measurement can be studied for the resealing of pores [236]. Depending on the skin’s occlusion and the geometry of the needle, it can take 2–40 h to recover [237]. TEWL and the shading of tissues can also be used for pores [238]. The MNs do not contact the pain receptors deep inside the dermis, causing less pain than the needle of a hypoderm [239]. The pain severity is influenced by the MN numbers on a patch, the MN duration, and the tip angle or needle shape [6]. The length and number of the MNs on the spot are smaller than the discomfort involved with the procedure [240,241].

## 8. Key Human Diseases Studied by Polymeric MNs

Polymeric MNs have been studied for the treatment of various diseases such as cancer, HIV, diabetes, etc. They have also been used for vaccination and immunization. A successful translation of MN technology requires careful consideration for the targeted disease. To motivate this point, we discuss some key diseases targeted for polymeric MNs application in this section. Furthermore, Table 3 summarizes different drug molecules administered through polymeric MNs.

Diabetes. Diabetes mellitus is a common metabolic disorder that involves hyperglycemia or an excess of glucose in the bloodstream due to insulin deficiency or abnormality in the use of insulin [242]. Early work on insulin delivery was performed by administering insulin using nanovesicles combined with iontophoresis. The transdermal administration through porcine skin was aided by a solid stainless-steel MN array that perforated the skin, creating microchannels. The nanovesicles combined with iontophoresis and MNs had a penetration rate of insulin 713.3 times higher than passive diffusion [243]. Darvishha and Amiri [244] reported the viability of swellable MNs for the purpose of delivering high molecular weight molecules, such as insulin. Darvishha and Amiri [244] describe how the MNs swell and dissolve when they are inserted into the skin, allowing diffusion of the drug from the MN into the skin. The authors also state that swellable MNs provide a transdermal route of delivery with a prominent level of control on the release of compounds from the MNs [244]. Vora et al. [245] discussed the potential of dissolving MNs manufactured from the carbohydrate biopolymer pullulan to deliver low and high molecular weight drugs. Vora et al. [245] determined that the pullulan dissolving MNs were able to penetrate porcine skin and successfully deliver the insulin encapsulated within the MNs. With the stability of the insulin encapsulated confirmed by circular dichroism, pullulan dissolving MNs were shown to provide a viable route of insulin delivery [245]. Research performed by Yu et al. [86] is of significant interest as well. Yu et al. [86] validated a glucose-responsive polymeric MN in the form of a “smart insulin patch” that controls blood glucose levels by altering the insulin released in accordance with the quantity of glucose within the blood. This alteration is due to function of dissolution kinetics of blood glucose [86,246].

**Figure 10:**
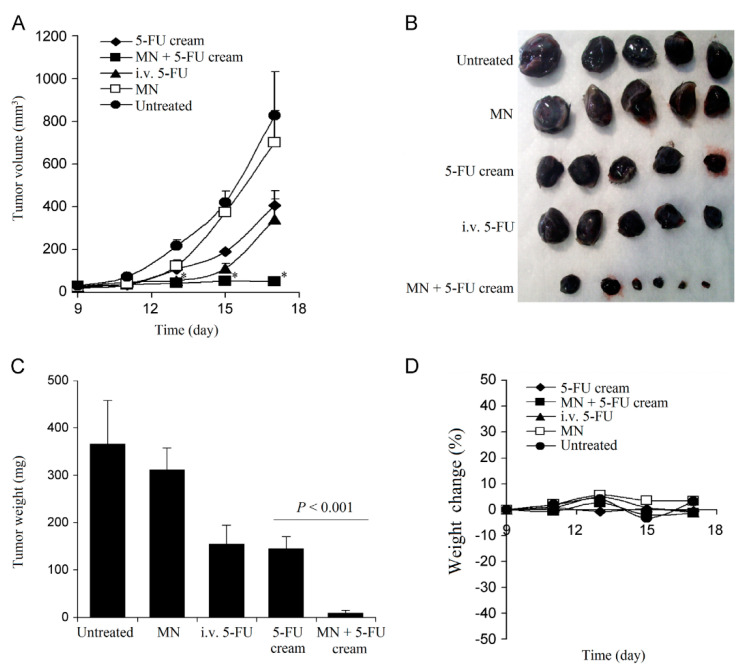
(**A**) The growth curves of B16-F10 tumors in mice. The asterisks (*) in (**A**) indicates that the values of the 5-FU cream group and the MN + 5-FU cream group are different on day 13, 15, and 17 (*P* < 0.05). (**B**) Digital photographs of tumors at the end of the study. (**C**) The weights of tumors at the end of the study. (**D**) The changes in the body weight of B16-F10 mice, reproduced with permission from [256], Elsevier, 2013.

**Figure 11:**
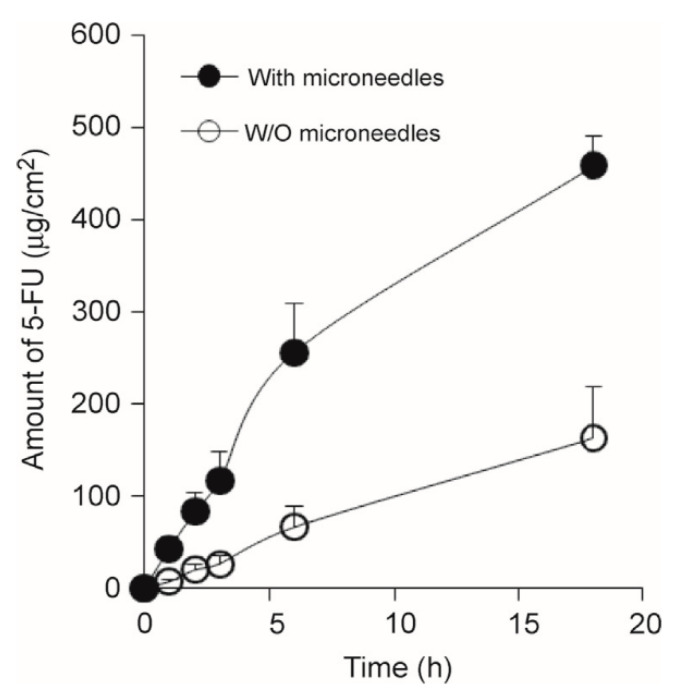
The amount of 5-FU in an aqueous solution diffused through full-thickness mouse skin treated (●) or not treated (◌) with MNs as a function of time, reproduced with permission from [256], Elsevier, 2013.

Basal Cell Carcinoma. Skin cancer is a worldwide threat [247]. Basal cell carcinoma (BCC) is a type of melanoma skin cancer caused by exposure to UV light from the sun or, more recently, sunbeds [248]. It can be found on the most exposed areas to the sun, such as the face, head, neck, and ears. Although it is not a hereditary disease, the people most susceptible to BCC are fair-skinned or those who have prolonged exposure to the sun [249]. Superficial BCC is a locally destructive skin tumor on the epidermis that can leave red scaly marks on the skin while other types can result in the formation of lumps [250]. If left untreated, BCC can lead to the development of skin ulcers that can be painful and itchy [251]. Although BCC is curable, the longer it is neglected, the more complex the treatment becomes [252]. Therapeutic techniques to treat BCC such as curettage, cautery, cryotherapy [253] exist, including the topical use of creams such as imiquimod (IMQ) and 5-fluorouracil (5-FU) as a noninvasive approach [254]. MNs have been introduced to improve the skin’s permeability of molecules as delivery efficiency is not optimized by the sole use of such creams [46]. Most of the drug remains in the formulation and is not delivered [255]. In an experiment conducted by Naguib et al. [256], it was found that the flux of 5-FU through the skin was increased by up to 4.5-fold when the skin was pretreated with the Dermaroller^®^ MN (500 µm in length, 50 µm in base diameter). Figure 10A,B [256] show the decreased visibly in size and Figure 10C,D [256] show the decreased weight of the tumor after MN treatment. B16-F10 murine melanoma cells (100,000 cells per mouse) in 100 µL DMEM were injected subcutaneously in the lower dorsal skin of anesthetized mice. To investigate the effect of MNs on the skin’s permeability and antitumor activity of topical 5-FU, the mice were randomly grouped: (1) mice treated with 5% 5-FU once a day for 8 consecutive days; (2) MN + cream group where mice were pretreated with MNs prior to application of the cream; (3) I.V. 5-FU the positive control group where mice were injected with 5-FU in sterile PBS intravenously via tail vein on days 9 and 15 of the experiment; (4) the negative control groups where tumor-bearing mice were left untreated; or (5) treated with MN only. Figure 11 illustrates the diffusion of aqueous 5-FU solution through the skin treated or not treated with MNs over time. Without MNs, the relationship between the amount of 5-FU diffused is almost linear; however, when treated with MNs, it is evident that the amount diffused in each time increased resulting in a steeper gradient. The use of statistical analysis on experimental data led to the conclusion that MNs can significantly (*p* < 0.05 two-tailed test) improve the in vitro skin permeability and in vivo anti-tumor activity of topical 5-FU [256].

HIV and Vaccination (Immunobiological Administration). In 2018, it was estimated that over 37.9 million people across the world had human immunodeficiency virus (HIV) [257]. HIV attacks the CD8+T cells of the body’s immune system, making it more difficult for the body to fight infectious diseases [258]. As a result, people with HIV become more susceptible to unintentional weight loss, chronic diarrhea, recurrent infections, and serious life-threatening illnesses, such as AIDS [259]. Vaccines can deliver protective antibody responses against HIV. Hence, the safe and effective delivery of HIV vaccines is imminent to minimize the impact of infection [260]. The skin has a high density of antigen cells in the epidermis and dermis, thus vaccines are typically administered through injections either intramuscular or subcutaneously, which have demonstrated effective systemic immunization [259]. However, exploration of the possible benefits of solid dosage forms over liquid dosage forms has led to the introduction of vaccine delivery via MNs [258]. Overall, solid dosage forms provide a higher level of immunogenicity, resulting in a dose-sparing effect and making vaccines more cost-effective [261]. With hollow polymeric MNs, the dose can be minimized, as seen with the rabies and anthrax vaccines [262,263]. The use of MNs also omits the use of cold-chain storage used for conventional vaccines due to better thermostability [28]. In addition, MNs are not limited in their applications for the delivery of different vaccinations. Coated MNs have been effectively tested to deliver Bacillus Calmette-Guerin (BCG) to guinea pigs for tuberculosis, resulting in a 1.3-fold higher IFN- γ in the lungs compared to a hypodermic needle [264].

Contraception—Transdermal Patches. While the introduction of the contraceptive pill in 1974 to the National Health Service (NHS), United Kingdom (UK) was pivotal to women’s health, the current state of reproductive and sexual health in the UK highlights the inconsistencies in contraception and effectiveness [265]. Although this cannot be determined solely by one factor, it does perpetuate the need for contraceptive innovation. One way to approach this is via transdermal delivery of contraceptives via penetration through the skin for systemic delivery [266]. The major concern with transdermal delivery is penetration affecting the pharmacokinetics of absorption; thus, the efficacy is influenced. Researchers are currently investigating MN skin patches for long-acting contraceptive delivery. Mofidfar et al. [80] aimed to create a self-administering long-acting contraceptive MN patch applied once a month for 5 s. The use of an effortless MN patch may combat several issues with the orally-administered contraceptive pill. The forgetfulness and, therefore, ineffectiveness may potentially be minimized; patients only must remember to apply it once per month. The increased level of estrogen exposure and reduced variability in plasma concentration of the pill minimizes typical side effects such as diarrhea, nausea, and vomiting. The patch also creates a hormone gradient, allowing a controlled and constant release rate [80,81]. Current patches are more than 99% effective (when used correctly) against pregnancies but do not protect against STIs such as less effective barrier devices in the same way as condoms. The number of drugs that can be integrated into an MN patch is limited and this technology is yet to be tested in humans as far as the authors are aware [267].

Dermatological Conditions and Cosmetics. The advances in the transdermal application of MNs to systemically deliver drugs through the skin have provoked the use of MNs for the treatment of dermatological conditions and nonmedical cosmetic therapeutics [268]. The application of MNs to treat acne, atrophic scars, actinic keratosis, hyperhidrosis, melasma, skin rejuvenation, alopecia, and more severe dermatological diseases can potentially be revolutionary in this field [269]. Dermatological conditions are proven to cause low self-esteem in individuals, leading to psychological problems [270]. In a recent study, MNs were combined with phototherapy to transmit light deeper into the skin, improving the use of phototherapy as a cosmetic and medical tool. The combination significantly increased the light transmissivity by 160%. For phototherapy with MNs, the light is internally reflected and then refracted at the tip of the MNs, mimicking optical fibers [271]. Such treatment can be used to treat psoriasis, a chronic papulosquamous disease estimated to affect 3% of the world’s population, according to the International Federation of Psoriasis Association [272]. It primarily affects the skin as well as nails, joints, and tendons. Acanthosis, increased thickness of the dermal layer, and hyperkeratosis of the SC are responsible for the scaly appearance and may cause the skin to be inflamed, bleed, crack, itch or shed scales. This is most found in areas such as the elbow, knee, and scalp [273]. There are a variety of treatment options for psoriasis, which are suppressive but of which none are curative [274]. Methotrexate is a drug that is typically administered orally or parenterally to help treat psoriasis. However, the side effects of systemic exposure to methotrexate include nausea, vomiting, and anemia [275]. Alternatively, Vemulapalli et al. [105] investigated the transdermal delivery of methotrexate utilizing MNs and iontophoresis. The solid maltose MN used to deliver Methotrexate with an applied voltage caused a 25-fold improvement of delivery into the skin in vivo [105]. Anti-TNF-α antibody therapy was employed to reduce the epidermal inflammation in psoriatic lesions. When anti-TNF-α was applied to a psoriatic mouse, the critical biomarkers of psoriasis inflammation were drastically reduced [276]. This suggests that with further research, the future application of MNs to treat psoriasis and other dermatological conditions of this nature can be widely improved. To demonstrate further the effect of combining MNs with techniques to treat dermatological conditions, Konicke and Olasz [277] investigated the use of a commercial Dermapen^®^ and topical bleomycin to treat plantar warts. Plantar warts are viral proliferations caused by human papillomavirus (HPV) infection [278]. Current practices involve the physical destruction of the infected cells via cryotherapy. The limitations of such treatments are the intense pain associated and the recurrence of warts [279]. This clinical study concluded that with the use of MNs and bleomycin, all patients were completely cured with minimal pain [280]. The cosmetic field is the most progressive in the day-to-day physical application of MNs [268]. MNs have surpassed trial phases and are now available in clinical practices [281]. It is more advantageous than other techniques such as dermabrasion, laser treatment, or chemical peeling as there is less damage to the epidermis [282]. It is a quick and safe procedure resulting in skin rejuvenation due to the induced production of collagen. Collagen regenerates the skin to look smoother and healthier [283]. This is particularly appreciated for aging skin because, with time, the skin becomes dehydrated, elasticity decreases, and it is continually thinning [271]. Hyaluronic acid MNs have been statistically proven (*p* < 0.05) to improve wrinkles and skin hydration. This is attributed to its high-water binding and uptake capacity, biocompatibility, and biodegradable properties, making it suitable for anti-wrinkle treatment [284]. The delivery of other cosmetic ingredients such as ascorbic acid, retinoic acid, and adenosine has also been investigated [9]. Adenosine-loaded dissolvable MNs show better efficacy and skin improvements than topical adenosine cream. Despite the dosage of the MNs being 140 times less (10.72 µg) than the cream (1400 µg), this underlines the significance of the SC as a chemical and physical barrier [271].

**Table 3:** Examples of polymeric MNs used in TDD.

Drug/Molecule Loading (Targeted Disease)	Polymeric Material *	In Vivo/In Vitro Analysis	Type of MN	Advantages/Key Results	References
Etonogestrel (Contraceptive hormone)	PVA, HPMC	Female Sprague-Dawley rats, (200 ± 20 g)	Dissolving	Sustained release for around 1 week Fabrication process was optimized to increase drug loading without compromising the mechanical strength of the MN	[285]
Levonorgestrel (Contraceptive hormone)	PLGA, PLA	Adult female Sprague-Dawley rats, (200 ± 12g)	Dissolving	Air bubble between MN and patch backing for easy removal Sustained-release was achieved, maintaining concentration above the human therapeutic level for 1 month	[80]
Bleomycin (Treatment of warts)	PLA	Porcine skin, (2.3318 ± 0.22 mm thick)	Coated	The MN had high mechanical strength and was able to transport 80% of bleomycin in 15 min Can be used at different anatomical sites	[286]
Poly hexamethylene biguanide (PHMB) (Ocular diseases)	PLGA	Eight-week-old female mice	Dissolving	Patch was applied to the cornea Sustained drug release was achieved for 9 days when applied to the cornea	[140]
Bevacizumab (Cancer)	PVA (MW: 9–10 kDa)	Female Sprague-Dawley rat	Swellable, dissolving	Successful in diffusing high molecular weight drug Bevacizumab (MW: 149 kDA) Targeted delivery of chemotherapeutic agents to the lymphatic system was achieved Comparable study performed on dissolving and swellable MN	[287]
Esketamine (Treatment-resistant depression)	PMVE/MA + PEG (Gantrez^®^)	Female Sprague-Dawley rats	Swellable	No polymer degradation in skin Parafilm M used as a substitute model for the MN insertion study Sustained delivery of Esketamine was achieved for >24 h.	[219]
Amyloid β peptide (Aβ) (Alzheimer’s disease)	MicroHyala (containing Hyaluronic acid)	APPPS1 mice (genetic background; C57BL/6)	Dissolving	Approved polymeric MN for wrinkle treatment 100% delivery of encapsulated Aβ with efficient immune response.	[288]
Rapamycin (Skin tumors and vascular anomalies)	PVP (MW: 10 kDa)	Female BALB/c mice	Dissolving	Improved delivery of poorly water-dissolvable rapamycin 80% of the drugs into the skin in 10 min.	[220]
Tetanus toxoid (Immunization)	PVA (MW: 160 kDa), PVP (MW: 30 kDa)	Swiss-Albino mouse	Dissolving	Complete dissolution within 1 h of insertion.	[190]
Doxorubicin (Cancer)	GelMA	Mouse cadaver skin	Dissolving	Possibility of tunable drug release by adjusting the crosslinking density of GelMA MN swells initially followed by enzymatic degradation Ability to administer macromolecules (e.g., protein, nucleic acid)	[289]
Penta gastrin, Sincalide (Therapeutic peptides)	PVP (MW: 40 kDa)	Freshly excised skin (porcine ear)	Dissolving	Capable of delivering several types of peptides Release rate varies with peptide’s physical and chemical properties Looked into the effect of MW on delivery of peptide.	[290]
Dihydroergotamine mesylate (Migraine)	PVA (MW: 6 kDa), PVP (MW: 10 kDa),	Male Sprague–Dawley rats (500–550 g)	Dissolving	The delivery was in line with the subcutaneous administration with high relative bioavailability (97%).	[291]
Ovalbumin (Vaccination)	PMVE/MA-PEG (Gantrez^®^)	BALB/c mice	Swellable	Highlighted the importance of MN design and composition on the immune response to vaccine antigens	[292]
Doxorubicin HCl and docetaxel (Cancer)	PVA (MW: 160 kDa), PVP (MW: 40 kDa)	Excised mouse skin	Dissolving	MNs dissolved within 1 h of insertion in excised skin Co-delivery of doxorubicin and docetaxel	[63]
Amlodipine (Hypertension)	PEEK LT-3 grade	Pig ears skin	Coated	FDA approved polymer Effects of various MN geometry parameters on the degree of drug permeability enhancement were studied	[293]
Tranexamic acid (Melasma)	PVP (MW: 40 kDa)	Anesthetized Albino rat	Dissolving	All loaded drugs were released within 7 h of insertion Simulation studies were performed to understand the drug delivery mechanism using COMSOL software	[294]
Gentamicin (Neonatal sepsis)	PVA (MW: 9–10 kDa), PVP (MW: 360 kDa), PEG (0.4 kDa)	Female Sprague-Dawley rats (208.65 ± 21.48 g)	Dissolving	Three different doses of Gentamicin were successfully delivered into the skin with a delivery time ranging from 1 to 6 h	[153]

* Reference: GelMA: gelatin meth acryloyl; HA: hyaluronic acid; HPMC: hydroxypropyl methylcellulose; PEEK: polyether ether ketone; PEG: polyethylene glycol; PLGA: poly (lactic-*co*-glycolic acid); PMVE/MA: polymethyl vinyl ether-alt-maleic anhydride; PVA: polyvinyl alcohol; PVP: polyvinylpyrrolidone.

## 9. Polymeric MN Based Devices and Preclinical and Clinical Trials

By the year 2025, the market for TDD is estimated to be worth approximately $95.57 billion [295]. Consequently, many preclinical and clinical trials have been conducted that involve polymeric MN devices. In the fabrication of an MN master template, it is required to have thousands of USD and the access and arrangement of facilities such as a clean room and photo-etching equipment for the implementation of techniques such as deep-reactive ion-etching, laser-etching, and anisotropic wet etching [296,297]. Hurdles are encountered along the way of clinical translation of this device [298]. The current production methods are often limiting as these methods employ batch production processes [297,299]. The biggest obstacle is in developing processes that allow for robust, efficient, and high throughput production in an industrial setting [31]. The usage of 3D printing technology for fabricating MNs can overcome the existing disadvantages of conventional techniques and can provide high precision, rapid fabrication, reduced processing steps, and freedom to print a wide range of shapes [160,161,162,166].

**Figure 12:**
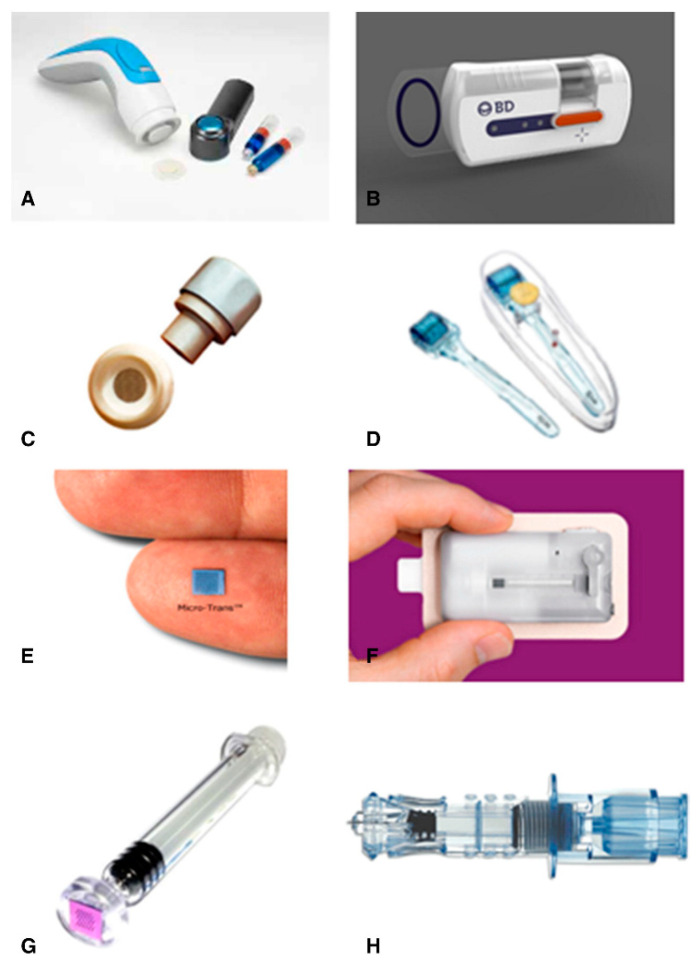
Current MN devices. (**A**) Micro structured Transdermal System, (**B**) Microinfusor, (**C**) Macroflux^®^, (**D**) MTS Roller™, (**E**) Micro-trans™, (**F**) h-patch™, (**G**) MicronJet, (**H**) Intanza^®^, reproduced with permission from [326], Elsevier, 2014.

MNs have important advantages relative to traditional transdermal pads, particularly in the delivery of biopharmaceuticals [300]. Thus, in the last decade, economic activity in this field has grown considerably [301]. Several MN-based drugs are currently being produced by various firms, including Zosano Pharma (USA), 3M (USA), Sanofi Pasteur MSD (USA), Becton-Dickinson (BD) Technologies (USA), Valeritas (USA), Nano pass Technologies (Israel), MN Therapy System (USA), and Rodan+ Fields (USA). Remarkably, no drug delivery product based on the MN array is yet commercialized [297]. Micro-injection systems are also a type of MN-based device being developed. There exists Micronjet^®^ (no-pass) and Soluvia^®^ (BD), but they are not genuine MN arrays and are instead small hollow needles that can be used to insert a traditional syringe barrel with effective ID. These devices work in the same manner as traditional syringes. MicronJet^®^, a hollow MN device for intradermal injection, was developed by Nano pass Technology. The device consists of four hollow silicon needles smaller than 500 μm, which are attached to a plastic device that can be connected to any regular syringe. This method was used to vaccinate influenza with the immunogenicity of at least 20% of the traditional vaccine dosage. In 2010, the FDA approval was granted to Micronjet^®^ [302,303]. The Micro structured Transdermal System^®^, which consists of coated MN arrays, was developed by 3M, which allows quicker delivery of medicines and vaccines. This machine demonstrated quick distribution, with a manageable administration for up to 90 min [304,305,306]. BD Technologies has produced a new type of machine, the Microinfusor. This machine is automatic and hands-free for delivery in a few seconds to several minutes of a large variety of pharmaceuticals to the subcutaneous tissue. The hollow MN device can be used to supply extremely viscous biotech medicines with a capacity of 0.2 to 15 mL [307,308,309,310]. Preclinical experiments found the delivery of the influenza vaccine with the same potency as a traditional intramuscular injection [146,222,235]. The Swiss business DE biotech has taken a similar approach. They also developed an injector system with one or more MNs called DebioJectTM silicon. It can be used to inject in under 2 s up to 100 μL and in less than 5 s up to 500 μL [311,312]. Sanofi Pasteur MSD Limited has already developed an intradermal influenza vaccine micro-injection system. This pioneering machine is called Intanza^®^ and uses the BD technologies Soluvia^®^ injector. This device includes a hypodermic needle 1.5 mm long attached to a syringe injector [214,305]. Soluvia^®^ and MicronJet^®^ are currently the only therapeutic-based MN-basic devices on the market. This injector is currently sold globally under the names IDflu^®^ and Fluzone Intradermal^®^ in addition to Intanza^®^ [313,314,315]. In the last few years, MN rollers have become available on the market [316,317,318]. The FDA has authorized the MTS RollerTM for cosmetics. Clinical trials have shown, in enhancing collagen and elastin development and eradicating wrinkles, that this type of device is more successful than other traditional ablative and Non-ablative therapies [268,319,320,321]. Two types of MN-based systems were developed by Valeritas. The first one was Micro-TransTM Array Patch, a system for painless delivery to dermatics. The second was the h-PatchTM, a tool for controlled subcutaneous drug delivery [322,323]. Alza Macroflux^®^ has been designed with coated titanium micro-projections as an optimized delivery of biopharmaceuticals. In this system, the skin penetration depth can be reproductively regulated due to the integration of an applicator device system. Moreover, the ovalbumin delivery system has been successfully tested [324]. Rodan+Fields Dermatologists produced a cosmetic MN product available on the market. It is a dissolving MN series of hydrolyzed hyaluronic acid for cosmetic use [325]. Furthermore, MN analysis was extended, and a more diverse and extensive MN product was created. Few currently available MN products are presented in Figure 12 [326].

Although polymeric MNs are biocompatible, when the accumulation of polymers occurs in our bodies through repeated application of the MN arrays, hepatic impairment, immunological reaction, and build-up of polymers in the dermal tissue are probable health implications with the use of this device [327]. One of the major challenges associated with the use of polymeric MNs is the penetration of MNs through the skin layer [328]. Thus, the insertion ability of the MN should not involve any bending or breakage [329]. To combat this challenge, a mixture of two or more polymers is used such that the mechanical strength and structural integrity are not compromised. It is important to strike the proper balance of flexibility and strength when targeting soft tissues that may not withstand the pressure of high-strength MN insertion [330].

To develop polymeric MNs, environmental factors need to be taken into consideration as well. The polymers used in MN fabrication can be hygroscopic [88]. This hygroscopic nature causes the arrays of MNs to absorb water from the production facility. The presence of moisture exerts a negative impact on the structural integrity and strength of the finished product. Eventually, the performance of the final MN product is also hampered [331]. Therefore, initiatives must be undertaken in the MN production facilities and methods necessary for the successful fabrication of MN arrays [332]. As the technology of MN-based products is quite innovative in terms of application in the clinical setting, sufficient standards are not available in the pharmacopeia in this regard [19]. There exists the need for the maintenance of environmental conditions for standardization and regulation regarding sterility, safety, durability, application, and disposal of MNs that will help in the commercialization of polymeric MNs in the pharmaceutical sector [106].

**Figure 13:**
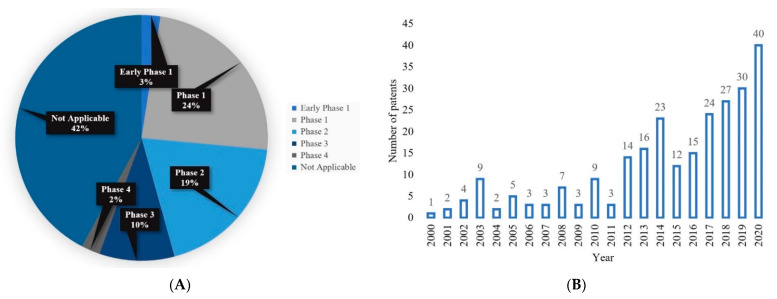
(**A**) Comparative percentage of completed MN clinical trials on ClinicalTrials.gov database. In total, 76 clinical trials data were found in distinct phases from the database. Information accessed on 21 February 2021 at www.clinicaltrials.gov, and (**B**) the number of issued US Patents for MN per year from 2000 to 2020. A total of 252 MN patents are available on the database. Information accessed on 21 February 2021 at http://patft.uspto.gov/.

There are several clinical trials related to MN technology available on the ClinicalTrials.gov database. Data from 116 clinical trials are available on the database, using the keyword “microneedle”. The selected number of clinical trial studies in this review was 76 based on the “complete” status (information accessed on 21 February 2021 at www.clinicaltrials.gov). Depending on the availability of data, an overview of the percent of MNs in the clinical stage state and selected clinical trials are listed in Figure 13A and Table 4, respectively. Initially, a total of 260 issued US patents were found from the United States Patent and Trademark Office database (Information accessed on 21 February 2021 at http://patft.uspto.gov/netahtml/PTO/search-bool.html) using the term “microneedle” and field “Title”. From there, specific to each year, 2000–2020 were searched and is presented in Figure 13B. A total of 252 issued patents were the sum from 2000 to 2020. Table 5 lists the US patents relevant to the polymeric MN system.

## 10. Regulatory Issues with Polymeric Microneedles

In the last two decades, a significant effort has persisted for the alternative use of MNs in clinical practice to be more concrete [354]. As researchers have become aware of the potential of MN technology, several organizations have taken steps to switch from laboratory-scale experiments to pre-commercial stages [137]. Cosmetics have been the simplest to work with MNs [355]. However, concerns have been raised in using MNs in clinical practices, such as in hospitals, regarding their sterility, reliability, and protection before disposal and after use [107]. The fundamental issues have been addressed hitherto, in which the criteria and limitations have relied significantly on implementation [356]. Several systemic approaches to these issues, especially with sterile MN production and stability, have been published in the literature. Sterilization is based on gamma-ray irradiation, as required by the European Pharmacopoeia [45]. Depending upon the form of the polymeric matrix, this process may affect polymeric MNs differently [357]. It has been argued that the least impacted during the sterilization process are the swellable MNs as compared to the dissolvable MNs [358]. The MNs secure the molecules loaded in the polymer from the point of view of medical stabilization [359]. During the cold chain and other climate pressures, these operations have been maintained for vaccines [360].

Studies on diabetes, psoriatic plaques, topical anesthetics, and influenza vaccines have involved hundreds of MN devices for clinical trials. Without laboratory trials with volunteer human subjects, clinical translation of the emerging medical innovations is unlikely to be successful. Dissolvable but also permanent, MNs prove to be easy to use and well-tolerated in clinical trials. Various study groups have recently published the results of MNs in dermatological disorders such as scars and keloids. In both cases, MNs have improvedimproved the efficiency of treatments by self-administration and painlessness. In several experiments, hollow MN systems that are commercially available were used, although clinical trials on polymer MNs are limited. The penetration potential and protection of the HA-base dissolving MNs have been investigated in two Phase I experiments. The findings indicate that the MNs can be reproducibly absorbed through the skin with the aid of an applicator and no apparent side effects. The protection and immunogenicity of dissolvable MNs dependent upon HA were analyzed in another phase trial. In the clinical trial for the delivery of strains of seasonal H1, H3N2, and B influenza virus vaccines, results revealed that the MN patch is tolerable, and a strong immune response is possible. These experiments have shown that polymer MNs can be translated clinically. However, we know that to date, no clinical trials on the continuous release of MNs are published [361,362,363,364,365,366]. Immune modeling and the treatment of diseases in mice models have been effectively developed and implemented by MNs with continued release properties of drugs or vaccines. Many have demonstrated superior strength with the standard quick-dissolution MNs or the possibility to use free drug and vaccine formulations as a patient-friendly alternative to conventional continuous release approaches [360,363].

Consequently, Because of its creativity and technologies, there are no regulatory criteria specified now for MN array-based products [367]. The different mechanisms of action, therefore, indicate that MN is known from a regulatory viewpoint as a modern dose form rather than a special pre-existing transdermal patching device. In Addition, the regulatory requirements should be specified for these new goods [368]. There are guidelines to help develop global specifications for emerging drugs and drug products not previously licensed in the United States, the European Union, or Japan [369]. The International Conference on Harmonization of Legal Criteria on Pharmaceuticals Classification for Human Use has issued guidelines, which is classified as Q6A [370]. According to these guidelines, the following requirements are specified: “List of tests, references to analytical methods, and acceptable standards to be approved for the tests mentioned, including numerical limits, range or other criteria” [63,208]. It shows the conditions to be deemed suitable for the planned usage of medicinal material or drug items. Specifications form an essential factor of the quality assurance system and are needed to ensure that medications and drug products of superior qualities are continuously manufactured [371,372]. In short, the main regulatory issues to be resolved for MN product requirements are as follows [95]:

1. The MN dosage forms microbiological requirements. In certain instances, the MN dose formulations pierce the SC to the dermis. Microbiological requirements can also represent a significant regulatory concern. If there is also a proven antimicrobial activity, it may be appropriate for a substance to have low bioburden [21,65,373,374];
2. Content standardization: this is a standard prerequisite for pharmacopeia to be used in MN schemes. This condition can, however, be extended to either the entire system or to individual drug-loaded MN in an array, depending on the system configuration [375,376];
3. Certain MN devices cannot be dissolved or biodegraded and can be re-inserted easily. This can be dangerous and requires a proper disposal process [377,378];
4. Certain MN chemicals accumulate on the skin. Polymeric MN dissolvable deposits can deposit materials, causing adverse effects on the skin, such as the formation of granulomas or local erythema. By alternating the application platform, this may be mitigated. As stated earlier, this problem must be solved, for the long-term use of MN goods [17,359,379,380,381].
5. Patient ease of use and reliability: without risks, patients should be able to use the goods as possible [77,84];
6. Correct delivery insertion: since MN injection does not induce discomfort or apparent emotion, the patient has no feedback. A proper program framework may be appropriate [381,382,383];
7. Repetitive use of immunological effects: repeated insertion of MN into the skin may cause immune reactions. The regulators should be assured of immunological protection [139,384,385];
8. Profile for long-term security: to date, human volunteers have conducted short-term security experiments. The long-term protection of the MN program should therefore be taken into consideration from the point of view of sporadic and repetitive demands [379,386,387].

## 11. Current Trends and Future Perspective

The combined efforts of engineering, pharmaceuticals, and immunology researchers can promote the future success of this technology and clinical translation [365]. A micro-based lithographic approach to the manufacturing of tapered-cone MNs was developed to create MNs using biodegradable sharp tips, micro-e-machining, and etching [26]. PDMS micro-molds have been produced, and a vacuum-based process for filling the forms with polylactic acid, polyglycolic acid, and their polymers was designed for replicating Microfabricated Master Structures [388]. The mechanical testing of the resulting needles measured the force that split the needles during axial loading and showed that this failed strength increased with the material and needle foundation diameter and decreased with the needle length [389]. The failure forces were normally much greater than the forces needed to inject MN into the skin, suggesting the mechanical properties of biodegradable polymers for MN were adequate [389]. Finally, the ranges of polymer MNs revealed that the permeability of human skin cadaver was improved by up to three orders of magnitude to a low-molecular tracer, a calcein, and a macromolecular, bovine serum Albumin [354]. These findings demonstrate that biodegradable polymer MNs can be produced with the right geometry and strength to inject them into the skin such that the transdermal transport of molecules can significantly increase [137].

Sustained drug release MNs demonstrated higher performance relative to fast dissolving MNs [107]. Nevertheless, certain MN parameters still need to be investigated or optimized in the future [356]. For their therapeutic transition, the limited drug loading potential of MNs is a limiting factor since their scale and volume are small [355]. Researchers have employed centrifugation, refilling, and evaporation of micro cardiogram enrichment with medicinal substances and drug-laden nanoparticles [45]. Another path toward improving the capacity of drug loading may be to develop larger MN patches or produce novel polymers with higher solubility or loading capacities for drug molecules [357]. Techniques used in MN preparedness often include a physical and chemical process that influences the structures or functionality of biotherapeutics [358]. New techniques are therefore important to mitigate the use of such extreme environments [359]. It also seems that there is a lack of systematic analysis on the effect of drug strength of the various release kinetics of the MN matrix. Several studies have shown that kinetics can be regulated and optimized by adjusting the MN matrix components or adjusting the chemical modification level of polymers. More mechanisms and studies of comparison must be detailed [360,363,390].

The effectiveness of polymer MNs relies heavily upon the polymer form or combination of polymers, their biocompatibility with drugs, their nature, and their mechanical ability [391]. Furthermore, several new improvements in the original technique used for microneedling has been made over the last 10 years [392]. In comparison to other surgical methods, this procedure can have better outcomes [91]. This is a particularly safe technique, in which the chance of post-inflammatory pigmentation with other epidermis damaging techniques is high [330]. With the invention of the MN delivery system, medical research is moving forward with the promise of versatility as traditional delivery mechanisms malfunction or cause patients undesired pain [393]. The fabrication of the medication delivery system for MNs is a big breakthrough into the world of medicine with the hope of numerous uses, in which traditional delivery methods malfunction or give patients unwanted discomfort [394]. Basic research and marketing campaigns should be paired with the acceleration of large-scale polymer MN development and with further understanding of the long-term adverse effects of polymers in regenerative medicine to provide guidelines for sterilization procedures [377].

The production of MN-based multifunctional smart bioprocessing is still desperately needed for potential biomedical applications, considering the impressive achievements around MNs [139]. It is believed that MNs will gradually and practically progress into mass production with the advancement of science and technology, adding comfort to people’s lives [233]. Researchers expect that in the future, these MN-based smart therapeutic devices will also be more desirable if long-term usage and multi-stage medication distribution are needed for health conditions such as chronic diseases, treatment of diabetes, and chronic pain management [395]. Present MN techniques should be improved to completely convert microchip-based MNs into clinical applications to reach broad-scale output. In the first instance, the vast majority of TDD systems focusing on MNs were concerned with the on-demand release of pharmaceutical goods, with minimal literature focusing on MNs with electrochemical or microchip-controlled sensors or drug release controls. To enhance programming and wireless networking technology, it is also important to change the precise and sensitive design of the electronic microchip sensing and effecting components. The perfect conditions with smart wearables should be such as this: body state should be tracked in real-time, and an early alert can be given by the MN wireless contact system to the patient’s physical unit until an alarming situation is detected. Then, a drug release system is activated, and the patient can be warned of this. To attain this goal, MNs combined with electronic microchip elements are of essential significance. In comparison, the present MN-based transdermal distribution primarily focuses on drug delivery, so MNs can, in the future, be used as cell therapy for local delivery [166,396,397,398,399].

**Figure 14:**
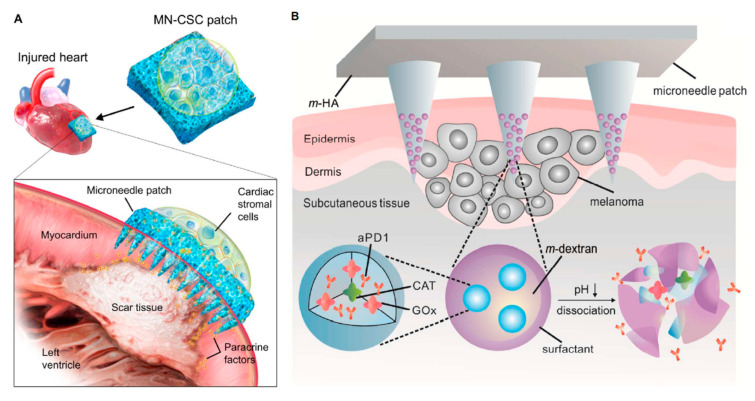
(**A**) Schematic illustrating the overall configuration used for measuring therapeutic benefits of infarcted heart microsomal cells. This groundbreaking work paves the way for the potential production for organ recovery and reconstruction of cell integrated MNs, reproduced with permission from [400], American Association for the Advancement of Science, 2018. (**B**) MN patch-assisted PD1 scheme for the prevention of skin cancer. Innovative projects pave the way for the potential creation of cancer immunotherapy focused on MNs, reproduced with permission from [401], American Chemical Society, 2016.

Researchers aim to make use of MNs to provide organ repair [400], as illustrated in Figure 14A, and regeneration with stem cells [84]. MNs can also be used as a depot of probiotics in the intestinal tract for the treatment of certain metabolisms to control intestinal microecology [78]. Certain essential questions must be resolved before reaching such an aim, such as how cell viability can be sustained and how cells inside the MN can be evenly distributed. Finally, the interest in immunotherapy has increased in recent years because of its high efficacy and its widespread use in several advanced biomedical fields [380]. There are also several studies of MNs being added to the provision of tumor control point inhibitors (shown in Figure 14B) [17,401]. Soon, we envisage using MNs not only to deliver immune inhibitors for the treatment of tumors but on-demand skin and tumor microenvironmental modulation as well as cytokine and immune cells [84,401]. Due to the recent developments in MNs, this target is possible [78]. MN-based immunotherapy is thought to be used in clinical practice by collaborative efforts of the multi-disciplined [381].

Despite major developments in the development, detection, and control of MNs for the delivery of transdermal drugs, biomedical applications are still early in existence and have not advanced toward large-scale manufacturing and practicality [355]. Shortly, these state-of-the-art developments may be combined with MNs [359]; however, they are not risk-free [378]. The most frequent side effect is mild inflammation of the skin after operation [402]. If the following signs exist: coughing, bruising, infections, peeling, etc., physicians should be advised [377]. It is necessary to look after the skin after an MN procedure for better outcomes, equivalent to any cosmetic treatment of the skin [403].

- Patients should not take anti-inflammatory drugs for a full week after treatment [404];
- Patients should not use ice on the face or use bromelain/arnica. This will impair a normal mechanism of inflammation that is essential to the rejuvenation of the skin [275,329];
- Prevention of sun tanning and extended direct sunshine use for at least 2 weeks. Use sunblock (30 SPF or higher) always after 24 h and wear a hat when outdoors [403];
- Patients with anxiety should take a painkiller or Tylenol [155].

## 12. Conclusions

This review paper demonstrates the value of polymeric MN as a promising technology, and it has a great future ahead. However, there are major obstacles in the immediate challenges of manufacturability, cost factors, and regulatory clearance. Science in this area is potentially advancing to safer supplies of transdermal drugs using the polymeric MNs method [405,406]. There has been noteworthy progress in the fields of transdermal medication using polymer MN [297]. With new polymeric substrates and manufacturing techniques [138,407,408], polymeric MNs can not only be administered upon request but can be individually customized to improve the quality of life of patients and achieve better therapeutic effects [107,161,399]. The creation of smart and compact MN devices is the general pattern of today, with the advancement of people’s living standards and the fast development of MNs [355]. Polymeric MN systems for the supply of medicines, diagnostic, thermo-therapeutic integrated, and wearable systems are the highlights in MN research [409]. 

## Figures and Tables

**Table 4:** List of completed clinical trials in MN technology. Information collected from the ClinicalTrials.gov database (Information accessed on 21 February 2021 at www.clinicaltrials.gov).

ClinicalTrials.gov Identifier	Title of Study	Investigating Condition	Clinical Trial Phase	Starting Year	Number of People	Location	Reference
NCT00837512	Insulin Delivery Using Microneedles in Type 1 Diabetes	type 1 diabetes mellitus	Phase 2 Phase 3	2008	16	United States	[333]
NCT01368796	Comparison of 4 Influenza Vaccines in Seniors (PCIRNRT09)	influenza vaccine	Phase 4	2011	953	Canada	[334]
NCT01812837	The Use of Microneedles in Photodynamic Therapy	actinic keratosis	N/A	2012	51	United States	[335]
NCT01674621	Phase 2 Study of BA058 (Abaloparatide) Transdermal Delivery in Postmenopausal Women with Osteoporosis	post-menopausal osteoporosis	Phase 2	2012	250	United States, Denmark, Estonia, Poland	[336]
NCT03203174	The Use of Microneedles with Topical Botulinum Toxin for Treatment of Palmar Hyperhidrosis	hyperhidrosis	Phase 1	2015	13	United States	[337]
NCT02438423	Inactivated Influenza Vaccine Delivered by Microneedle Patch or by Hypodermic Needle	influenza	Phase 1	2015	100	United States	[338]
NCT02745392	Safety and Efficacy of ZP-Zolmitriptan Intracutaneous Microneedle Systems for the Acute Treatment of Migraine (Zotrip)	acute migraine	Phase 2 Phase 3	2016	365	United States	[339]
NCT03282227	A Study to Evaluate the Long-Term Safety of M207 in the Acute Treatment of Migraine (ADAM)	migraine	Phase 3	2017	342	United States	[340]
NCT03739398	A Study on the Effectiveness and Safety Evaluation of Combination Therapy With 1927 nm Thulium Laser and Fractional Microneedle Radiofrequency Equipment for Improvement of Skin Aging	wrinkle	N/A	2018	26	Republic of Korea	[341]
NCT04253418	Nano-Pulse Stimulation (NPS) in Sebaceous Hyperplasia Optimization Study	sebaceous hyperplasia; skin abnormalities; skin lesion	N/A	2019	125	United States	[342]
NCT04249115	Nano-Pulse Stimulation (NPS) in Seborrheic Keratosis Optimization Study	lesion skin; seborrheic keratosis; skin lesion; benign skin tumor	N/A	2019	175	United States	[343]

**Table 5:** List of US patents related to polymeric MN technology. Information collected from the United State Patent and Trademark Office database (Information accessed on 21 February 2021 at http://patft.uspto.gov/netahtml/PTO/search-bool.html).

US Patents Number	Title	Issue Date	Inventor(s)	References
10737083	Bioactive components conjugated to dissolvable substrates of microneedle arrays	11 August 2020	Falo, Jr.; Louis D., Erdos; Geza	[344]
10682504	Microneedle and method for manufacturing microneedle	16 June 2020	Kato; Hiroyuki	[345]
10377062	Microneedle arrays formed from polymer films	13 August 2019	Kaspar; Roger L., Speaker; Tycho	[346]
10195410	Fabrication process of phase-transition microneedle patch	5 February 2019	Jin; Tuo	[347]
9498524	Method of vaccine delivery via microneedle arrays	22 November 2016	Corium International, Inc.	[348]
9302903	Microneedle devices and production thereof	5 April 2016	Park; Jung-Hwan, Prausnitz; Mark R.	[349]
8834423	Dissolvable microneedle arrays for transdermal delivery to human skin	16 September 2014	Falo, Jr.; Louis D., Erdos; Geza, Ozdoganlar; O. Burak	[350]
8708966	Microneedle devices and methods of manufacture and use thereof	29 April 2014	Allen; Mark G., Prausnitz; Mark R., McAllister; Devin V., Cros; Florent Paul Marcel	[351]
8366677	Microneedle arrays formed from polymer films	5 February 2013	Kaspar; Roger L., Speaker; Tycho	[352]
7429333	Method for fabricating microneedle array and method for fabricating embossing mold of microneedle array	30 September 2008	Chiou; Jin-Chern, Hung; Chen-Chun, Chang; Chih-Wei	[353]

## Data Availability

There are no additional raw data for this paper. The paper only uses secondary data from published papers and all credits to these data have been made via citations and copyright permissions.

## References

[1] Ripolin A., Quinn J., Larrañeta E., Vicente-Perez E.M., Barry J., Donnelly R.F. (2017). Successful application of large microneedle patches by human volunteers. Int. J. Pharm..

[2] Uddin J., Scoutaris N., Economidou S.N., Giraud C., Chowdhry B.Z., Donnelly R., Douroumis D. (2020). 3D printed microneedles for anticancer therapy of skin tumours. Mater. Sci. Eng. C.

[3] Peña-Juárez M.C., Guadarrama-Escobar O.R., Escobar-Chávez J.J. (2021). Transdermal Delivery Systems for Biomolecules. J. Pharm. Innov..

[4] Mercuri M., Rivas D.F. (2021). Challenges, and opportunities for small volumes delivery into the skin. Biomicrofluidics.

[5] Korenman S.G., Viosca S., Garza D., Guralnik M., Place V., Campbell P., Davis S.S. (1987). Androgen therapy of hypogonadal men with transscrotal testosterone systems. Am. J. Med..

[6] Pawasauskas J., Perdrizet G. (2014). Daily Application of Transdermal Fentanyl Patches in Patients Receiving Hyperbaric Oxygen Therapy. J. Pain Palliat. Care Pharmacother..

[7] Rouphael N.G., Paine M., Mosley R., Henry S., McAllister D.V., Kalluri H., Pewin W., Frew P.M., Yu T., Thornburg N. (2017). The safety, immunogenicity, and acceptability of inactivated influenza vaccine delivered by microneedle patch (TIV-MNP 2015): A randomised, partly blinded, placebo-controlled, phase 1 trial. Lancet.

[8] Prausnitz M.R. (2004). Microneedles for transdermal drug delivery. Adv. Drug Deliv. Rev..

[9] Waghule T., Singhvi G., Dubey S.K., Pandey M.M., Gupta G., Singh M., Dua K. (2019). Microneedles: A smart approach and increasing potential for transdermal drug delivery system. Biomed. Pharmacother..

[10] Calcutt J.J., Roberts M.S., Anissimov Y.G. (2020). Modeling drug transport within the viable skin—A review. Expert Opin. Drug Metab. Toxicol..

[11] Lin W., Cormier M., Samiee A., Griffin A., Johnson B., Teng C., Hardee G.E., Daddona P.E. (2001). Transdermal Delivery of Antisense Oligonucleotides with Microprojection Patch (macroflux^®^) Technology. Pharm. Res..

[12] Chen B., Wei J., Iliescu C. (2010). Sonophoretic enhanced microneedles array (SEMA)—Improving the efficiency of transdermal drug delivery. Sens. Actuators B Chem..

[13] Naik A., Kalia Y., Guy R. (2000). Transdermal drug delivery: Overcoming the skin’s barrier function. Pharm. Sci. Technol. Today.

[14] Larrañeta E., Lutton R., Woolfson A.D., Donnelly R.F. (2016). Microneedle arrays as transdermal and intradermal drug delivery systems: Materials science, manufacture, and commercial development. Mater. Sci. Eng. R Rep..

[15] Kusama S., Sato K., Matsui Y., Kimura N., Abe H., Yoshida S., Nishizawa M. (2021). Transdermal electroosmotic flow generated by a porous microneedle array patch. Nat. Commun..

[16] Yang S.-J., Jeong J.-O., Lim Y.-M., Park J.-S. (2021). Synthesis, and characterization of PVP microneedle patch using metal bioelectrodes for novel drug delivery system. Mater. Des..

[17] Hao Y., Li W., Zhou X., Yang F., Qian Z. (2017). Microneedles-Based Transdermal Drug Delivery Systems: A Review. J. Biomed. Nanotechnol..

[18] Parhi R.N.D.S. (2019). Review of Microneedle based Transdermal Drug Delivery Systems. Int. J. Pharm. Sci. Nanotechnol..

[19] Duarah S., Sharma M., Wen J. (2019). Recent advances in microneedle-based drug delivery: Special emphasis on its use in paediatric population. Eur. J. Pharm. Biopharm..

[20] Guillot A.J., Cordeiro A.S., Donnelly R.F., Montesinos M.C., Garrigues T.M., Melero A. (2020). Microneedle-Based Delivery: An Overview of Current Applications and Trends. Pharmacology.

[21] Chavoshi S., Rabiee M., Rafizadeh M., Rabiee N., Shamsabadi A.S., Bagherzadeh M., Salarian R., Tahriri M., Tayebi L. (2019). Mathematical modeling of drug release from biodegradable polymeric microneedles. Bio-Design Manuf..

[22] Kim K.S., Ita K., Simon L. (2015). Modelling of dissolving microneedles for transdermal drug delivery: Theoretical and experimental aspects. Eur. J. Pharm. Sci..

[23] Vora L.K., Moffatt K., Tekko I.A., Paredes A.J., Volpe-Zanutto F., Mishra D., Peng K., Thakur R.R.S., Donnelly R.F. (2021). Microneedle array systems for long-acting drug delivery. Eur. J. Pharm. Biopharm..

[24] Yadav P.R., Han T., Olatunji O., Pattanayek S.K., Das D.B. (2020). Mathematical Modelling, Simulation and Optimisation of Microneedles for Transdermal Drug Delivery: Trends and Progress. Pharmacology.

[25] Demir Y.K., Metin A., Şatıroğlu B., Solmaz M.E., Kayser V., Mäder K. (2017). Poly (methyl vinyl ether-*co*-maleic acid)—Pectin based hydrogel-forming systems: Gel, film, and microneedles. Eur. J. Pharm. Biopharm..

[26] Zhao X., Li X., Zhang P., Du J., Wang Y. (2018). Tip-loaded fast-dissolving microneedle patches for photodynamic therapy of subcutaneous tumor. J. Control. Release.

[27] Dathathri E., Lal S., Mittal M., Thakur G., De S. (2019). Fabrication of low-cost composite polymer-based micro needle patch for transdermal drug delivery. Appl. Nanosci..

[28] Jahan N., Archie S.R., Al Shoyaib A., Kabir N., Cheung K. (2019). Recent Approaches for Solid Dose Vaccine Delivery. Sci. Pharm..

[29] Sabri A.H., Kim Y., Marlow M., Scurr D., Segal J., Banga A.K., Kagan L., Lee J.B. (2020). Intradermal and transdermal drug delivery using microneedles–Fabrication, performance evaluation and application to lymphatic delivery. Adv. Drug Deliv. Rev..

[30] Eltayib E., Brady A.J., Caffarel-Salvador E., Gonzalez-Vazquez P., Alkilani A.Z., McCarthy H.O., McElnay J.C., Donnelly R.F. (2016). Hydrogel-forming microneedle arrays: Potential for use in minimally-invasive lithium monitoring. Eur. J. Pharm. Biopharm..

[31] Jamaledin R., Di Natale C., Onesto V., Taraghdari Z.B., Zare E.N., Makvandi P., Vecchione R., Netti P.A. (2020). Progress in Microneedle-Mediated Protein Delivery. J. Clin. Med..

[32] Ronnander P., Simon L., Koch A. (2020). Experimental and mathematical study of the transdermal delivery of sumatriptan succinate from polyvinylpyrrolidone-based microneedles. Eur. J. Pharm. Biopharm..

[33] Lee M.-T., Lee I.-C., Tsai S.-W., Chen C.-H., Wu M.-H., Juang Y.-J. (2017). Spin coating of polymer solution on polydimethylsiloxane mold for fabrication of microneedle patch. J. Taiwan Inst. Chem. Eng..

[34] Chen B.Z., Ashfaq M., Zhang X.P., Zhang J.N., Guo X.D. (2017). In vitro and in vivo assessment of polymer microneedles for controlled transdermal drug delivery. J. Drug Target..

[35] Leone M., Mönkäre J., Bouwstra J.A., Kersten G. (2017). Dissolving Microneedle Patches for Dermal Vaccination. Pharm. Res..

[36] Donnelly R.F., Majithiya R., Singh R.R.T., Morrow D.I.J., Garland M.J., Demir Y.K., Migalska K., Ryan E., Gillen D., Scott C.J. (2010). Design, Optimization and Characterisation of Polymeric Microneedle Arrays Prepared by a Novel Laser-Based Micromoulding Technique. Pharm. Res..

[37] Lee K., Lee J., Lee S.G., Park S., Yang D.S., Lee J.-J., Khademhosseini A., Kim Y.J., Ryu W. (2020). Microneedle drug eluting balloon for enhanced drug delivery to vascular tissue. J. Control. Release.

[38] Lahiji S.F., Kim Y., Kang G., Kim S., Lee S., Jung H. (2019). Tissue Interlocking Dissolving Microneedles for Accurate and Efficient Transdermal Delivery of Biomolecules. Sci. Rep..

[39] Yadav P.R., Pattanayek S.K., Chandra P., Pandey L.M. (2020). Modulation of Physicochemical Properties of Polymers for Effective Insulin Delivery Systems. Biointerface Engineering: Prospects in Medical Diagnostics and Drug Delivery.

[40] Wang M., Hu L., Xu C. (2017). Recent advances in the design of polymeric microneedles for transdermal drug delivery and biosensing. Lab Chip.

[41] Ye Y., Yu J., Wen D., Kahkoska A.R., Gu Z. (2018). Polymeric microneedles for transdermal protein delivery. Adv. Drug Deliv. Rev..

[42] Lopez-Ramirez M.A., Soto F., Wang C., Rueda R., Shukla S., Silva-Lopez C., Kupor D., McBride D.A., Pokorski J.K., Nourhani A. (2019). Built-In Active Microneedle Patch with Enhanced Autonomous Drug Delivery. Adv. Mater..

[43] Chiang H., Yu M., Aksit A., Wang W., Stern-Shavit S., Kysar J.W., Lalwani A.K. (2020). 3D-Printed Microneedles Create Precise Perforations in Human Round Window Membrane In Situ. Otol. Neurotol..

[44] Arshad M.S., Hassan S., Hussain A., Abbas N., Kucuk I., Nazari K., Ali R., Ramzan S., Alqahtani A., Andriotis E.G. (2019). Improved transdermal delivery of cetirizine hydrochloride using polymeric microneedles. DARU J. Pharm. Sci..

[45] Badnikar K., Jayadevi S.N., Pahal S., Sripada S., Nayak M.M., Vemula P.K., Subrahmanyam D.N. (2020). Generic Molding Platform for Simple, Low-Cost Fabrication of Polymeric Microneedles. Macromol. Mater. Eng..

[46] Sabri A.H., Cater Z., Gurnani P., Ogilvie J., Segal J., Scurr D.J., Marlow M. (2020). Intradermal delivery of imiquimod using polymeric microneedles for basal cell carcinoma. Int. J. Pharm..

[47] Than A., Liu C., Chang H., Duong P.K., Cheung C.M.G., Xu C., Wang X., Chen P. (2018). Self-implantable double-layered micro-drug-reservoirs for efficient and controlled ocular drug delivery. Nat. Commun..

[48] Hao Y.Y., Yang Y., Li Q.Y., Zhang X.P., Shen C.B., Zhang C., Cui Y., Guo X.D. (2020). Effect of polymer microneedle pre-treatment on drug distributions in the skin in vivo. J. Drug Target..

[49] Thakur R.R.S., Tekko I., Al-Shammari F., Ali A.A., McCarthy H., Donnelly R. (2016). Rapidly dissolving polymeric microneedles for minimally invasive intraocular drug delivery. Drug Deliv. Transl. Res..

[50] Amer R.I., Elosaily G., Bakr R.O., El Dine R.S., Fayez A. (2020). Characterization and Pharmacological Evaluation of Anti-Cellulite Herbal Product(s) Encapsulated in 3D-Fabricated Polymeric Microneedles. Sci. Rep..

[51] Moussi K., Bukhamsin A., Hidalgo T., Kosel J. (2020). Biocompatible 3D Printed Microneedles for Transdermal, Intradermal, and Percutaneous Applications. Adv. Eng. Mater..

[52] Luzuriaga M.A., Berry D.R., Reagan J.C., Smaldone R.A., Gassensmith J.J. (2018). Biodegradable 3D printed polymer microneedles for transdermal drug delivery. Lab Chip.

[53] Chen C.-H., Shyu V.B.-H., Chen C.-T. (2018). Dissolving Microneedle Patches for Transdermal Insulin Delivery in Diabetic Mice: Potential for Clinical Applications. Materials.

[54] Wang Q.L., Zhu D.D., Liu X.B., Chen B.Z., Guo X.D. (2016). Microneedles with Controlled Bubble Sizes and Drug Distributions for Efficient Transdermal Drug Delivery. Sci. Rep..

[55] Yang S., Wu F., Liu J., Fan G., Welsh W.J., Zhu H., Jin T. (2015). Phase-Transition Microneedle Patches for Efficient and Accurate Transdermal Delivery of Insulin. Adv. Funct. Mater..

[56] Donnelly R.F., Singh R.R.T., Alkilani A.Z., McCrudden M.T., O’Neill S., O’Mahony C., Armstrong K., McLoone N., Kole P., Woolfson A.D. (2013). Hydrogel-forming microneedle arrays exhibit antimicrobial properties: Potential for enhanced patient safety. Int. J. Pharm..

[57] Bediz B., Korkmaz E., Khilwani R., Donahue C., Erdos G., Falo L.D., Ozdoganlar O.B. (2013). Dissolvable Microneedle Arrays for Intradermal Delivery of Biologics: Fabrication and Application. Pharm. Res..

[58] Alizadeh-Osgouei M., Li Y., Wen C. (2019). A comprehensive review of biodegradable synthetic polymer-ceramic composites and their manufacture for biomedical applications. Bioact. Mater..

[59] Choi S.Y., Kwon H.J., Ahn G.R., Ko E.J., Yoo K.H., Kim B.J., Lee C., Kim D. (2017). Hyaluronic acid microneedle patch for the improvement of crow’s feet wrinkles. Dermatol. Ther..

[60] Chen M.-C., Wang K.-W., Chen D.-H., Ling M.-H., Liu C.-Y. (2015). Remotely triggered release of small molecules from LaB6@SiO2-loaded polycaprolactone microneedles. Acta Biomater..

[61] Nair L.S., Laurencin C.T. (2007). Biodegradable polymers as biomaterials. Prog. Polym. Sci..

[62] Dardano P., Caliò A., Di Palma V., Bevilacqua M.F., Di Matteo A., De Stefano L. (2015). A Photolithographic Approach to Polymeric Microneedles Array Fabrication. Materials.

[63] Bhatnagar S., Bankar N.G., Kulkarni M.V., Venuganti V.V.K. (2019). Dissolvable microneedle patch containing doxorubicin and docetaxel is effective in 4T1 xenografted breast cancer mouse model. Int. J. Pharm..

[64] Park J.-H., Allen M.G., Prausnitz M.R. (2007). Biodegradable polymer microneedles: Fabrication, mechanics and transdermal drug delivery. J. Control. Release.

[65] Chang K.-T., Shen Y.-K., Fan F.-Y., Lin Y., Kang S.-C. (2020). Optimal design and fabrication of a microneedle arrays patch. J. Manuf. Process..

[66] DeMuth P.C., Li A.V., Abbink P., Liu J., Li H., Stanley K.A., Smith K.M., LaVine C.L., Seaman M.S., Kramer J.A. (2013). Vaccine delivery with microneedle skin patches in nonhuman primates. Nat. Biotechnol..

[67] Tyler B., Gullotti D., Mangraviti A., Utsuki T., Brem H. (2016). Polylactic acid (PLA) controlled delivery carriers for biomedical applications. Adv. Drug Deliv. Rev..

[68] Nayak A., Das D.B. (2013). Potential of biodegradable microneedles as a transdermal delivery vehicle for lidocaine. Biotechnol. Lett..

[69] Chen M.-C., Ling M.-H., Kusuma S.J. (2015). Poly-γ-glutamic acid microneedles with a supporting structure design as a potential tool for transdermal delivery of insulin. Acta Biomater..

[70] Kim J.D., Kim M., Yang H., Lee K., Jung H. (2013). Droplet-born air blowing: Novel dissolving microneedle fabrication. J. Control. Release.

[71] Ito Y., Inagaki Y., Kobuchi S., Takada K., Sakaeda T. (2016). Therapeutic Drug Monitoring of Vancomycin in Dermal Interstitial Fluid Using Dissolving Microneedles. Int. J. Med. Sci..

[72] Ito Y., Yoshimitsu J.-I., Shiroyama K., Sugioka N., Takada K. (2006). Self-dissolving microneedles for the percutaneous absorption of EPO in mice. J. Drug Target..

[73] Ito Y., Hagiwara E., Saeki A., Sugioka N., Takada K. (2006). Feasibility of microneedles for percutaneous absorption of insulin. Eur. J. Pharm. Sci..

[74] Leone M., Romeijn S., Slütter B., O’Mahony C., Kersten G., Bouwstra J.A. (2020). Hyaluronan molecular weight: Effects on dissolution time of dissolving microneedles in the skin and on immunogenicity of antigen. Eur. J. Pharm. Sci..

[75] Lee H., Choi T.K., Lee Y.B., Cho H.R., Ghaffari R., Wang L., Choi H.J., Chung T.D., Lu N., Hyeon T. (2016). A graphene-based electrochemical device with thermoresponsive microneedles for diabetes monitoring and therapy. Nat. Nanotechnol..

[76] Martin C., Allender C., Brain K., Morrissey A., Birchall J. (2012). Low temperature fabrication of biodegradable sugar glass microneedles for transdermal drug delivery applications. J. Control. Release.

[77] Johnson A.R., Caudill C.L., Tumbleston J.R., Bloomquist C., Moga K.A., Ermoshkin A., Shirvanyants D., Mecham S.J., Luft J.C., DeSimone J.M. (2016). Single-Step Fabrication of Computationally Designed Microneedles by Continuous Liquid Interface Production. PLoS ONE.

[78] Huang D., Deng M., Kuang S. (2019). Polymeric Carriers for Controlled Drug Delivery in Obesity Treatment. Trends Endocrinol. Metab..

[79] Watanabe T., Hagino K., Sato T. (2014). Evaluation of the effect of polymeric microneedle arrays of varying geometries in combination with a high-velocity applicator on skin permeability and irritation. Biomed. Microdev..

[80] Li W., Terry R.N., Tang J., Feng M.R., Schwendeman S.P., Prausnitz M.R. (2019). Rapidly separable microneedle patch for the sustained release of a contraceptive. Nat. Biomed. Eng..

[81] Li W., Tang J., Terry R.N., Li S., Brunie A., Callahan R.L., Noel R.K., Rodríguez C.A., Schwendeman S.P., Prausnitz M.R. (2019). Long-acting reversible contraception by effervescent microneedle patch. Sci. Adv..

[82] Kochhar J.S., Quek T.C., Soon W.J., Choi J., Zou S., Kang L. (2013). Effect of Microneedle Geometry and Supporting Substrate on Microneedle Array Penetration into Skin. J. Pharm. Sci..

[83] Leone M., Priester M.I., Romeijn S., Nejadnik M.R., Mönkäre J., O’Mahony C., Jiskoot W., Kersten G., Bouwstra J.A. (2019). Hyaluronan-based dissolving microneedles with high antigen content for intradermal vaccination: Formulation, physicochemical characterization and immunogenicity assessment. Eur. J. Pharm. Biopharm..

[84] Hardy J., Larrañeta E., Donnelly R., McGoldrick N., Migalska K., McCrudden M.T.C., Irwin N.J., Donnelly L., McCoy C.P. (2016). Hydrogel-Forming Microneedle Arrays Made from Light-Responsive Materials for On-Demand Transdermal Drug Delivery. Mol. Pharm..

[85] Yang S.Y., O’Cearbhaill E., Sisk G.C., Park K.M., Cho W.K., Villiger M., Bouma B.E., Pomahac B., Karp J.M. (2013). A bio-inspired swellable microneedle adhesive for mechanical interlocking with tissue. Nat. Commun..

[86] Yu J., Zhang Y., Ye Y., DiSanto R., Sun W., Ranson D., Ligler F.S., Buse J., Gu Z. (2015). Microneedle-array patches loaded with hypoxia-sensitive vesicles provide fast glucose-responsive insulin delivery. Proc. Natl. Acad. Sci. USA.

[87] Singh P., Carrier A., Chen Y., Lin S., Wang J., Cui S., Zhang X. (2019). Polymeric microneedles for controlled transdermal drug delivery. J. Control. Release.

[88] Khan S., Minhas M.U., Tekko I.A., Donnelly R., Thakur R.R.S. (2019). Evaluation of microneedles-assisted in situ depot forming poloxamer gels for sustained transdermal drug delivery. Drug Deliv. Transl. Res..

[89] Chen M.-C., Lin Z.-W., Ling M.-H. (2015). Near-Infrared Light-Activatable Microneedle System for Treating Superficial Tumors by Combination of Chemotherapy and Photothermal Therapy. ACS Nano.

[90] Ke C.-J., Lin Y.-J., Hu Y.-C., Chiang W.-L., Chen K.-J., Yang W.-C., Liu H.-L., Fu C.-C., Sung H.-W. (2012). Multidrug release based on microneedle arrays filled with pH-responsive PLGA hollow microspheres. Biomaterials.

[91] Ullah A., Choi H.J., Jang M., An S., Kim G.M. (2020). Smart Microneedles with Porous Polymer Layer for Glucose-Responsive Insulin Delivery. Pharmaceutics.

[92] Cheng A., Sun W., Xing M., Zhang S., Gao Y. (2020). The hygroscopicity of polymer microneedles on the performance of dissolving behavior for transdermal delivery. Int. J. Polym. Mater..

[93] Koh K.J., Liu Y., Lim S.H., Loh X.J., Kang L., Lim C.Y., Phua K.K.L. (2018). Formulation, characterization and evaluation of mRNA-loaded dissolvable polymeric microneedles (RNApatch). Sci. Rep..

[94] Ng L.C., Gupta M. (2020). Transdermal drug delivery systems in diabetes management: A review. Asian J. Pharm. Sci..

[95] Lutton R., Moore J., Larrañeta E., Ligett S., Woolfson A.D., Donnelly R.F. (2015). Microneedle characterisation: The need for universal acceptance criteria and GMP specifications when moving towards commercialisation. Drug Deliv. Transl. Res..

[96] Gill H.S., Prausnitz M.R. (2007). Coated microneedles for transdermal delivery. J. Control. Release.

[97] Torrisi B., Zarnitsyn V., Prausnitz M., Anstey A., Gateley C., Birchall J., Coulman S. (2013). Pocketed microneedles for rapid delivery of a liquid-state botulinum toxin A formulation into human skin. J. Control. Release.

[98] FDA Regulatory Considerations for Microneedling Products. https://www.fda.gov/regulatory-information/search-fda-guidance-documents/regulatory-considerations-microneedling-products.

[99] Loizidou E.Z., Inoue N.T., Ashton-Barnett J., Barrow D.A., Allender C.J. (2016). Evaluation of geometrical effects of microneedles on skin penetration by CT scan and finite element analysis. Eur. J. Pharm. Biopharm..

[100] Lee J.W., Gadiraju P., Park J.-H., Allen M.G., Prausnitz M.R. (2011). Microsecond thermal ablation of skin for transdermal drug delivery. J. Control. Release.

[101] Lee J.W., Park J.-H., Prausnitz M.R. (2008). Dissolving microneedles for transdermal drug delivery. Biomaterials.

[102] Chen M.-C., Ling M.-H., Lai K.-Y., Pramudityo E. (2012). Chitosan Microneedle Patches for Sustained Transdermal Delivery of Macromolecules. Biomacromolecules.

[103] Khanna P., Luongo K., Strom A.J., Bhansali S. (2010). Sharpening of hollow silicon microneedles to reduce skin penetration force. J. Micromech. Microeng..

[104] Olatunji O., Das D.B., Garland M.J., Belaid L., Donnelly R. (2013). Influence of Array Interspacing on the Force Required for Successful Microneedle Skin Penetration: Theoretical and Practical Approaches. J. Pharm. Sci..

[105] Vemulapalli V., Bai Y., Kalluri H., Herwadkar A., Kim H., Davis S.P., Friden P.M., Banga A.K. (2012). In Vivo Iontophoretic Delivery of Salmon Calcitonin Across Microporated Skin. J. Pharm. Sci..

[106] Lee K.J., Jeong S.S., Roh D.H., Kim D.Y., Choi H.-K., Lee E.H. (2020). A practical guide to the development of microneedle systems—In clinical trials or on the market. Int. J. Pharm..

[107] Vicente-Perez E.M., Larrañeta E., McCrudden M.T., Kissenpfennig A., Hegarty S., McCarthy H., Donnelly R.F. (2017). Repeat application of microneedles does not alter skin appearance or barrier function and causes no measurable disturbance of serum biomarkers of infection, inflammation or immunity in mice in vivo. Eur. J. Pharm. Biopharm..

[108] Prausnitz M.R., Langer R. (2008). Transdermal drug delivery. Nat. Biotechnol..

[109] Ita K. (2015). Transdermal Delivery of Drugs with Microneedles—Potential and Challenges. Pharmaceutics.

[110] Williams A., Barry B.W. (2012). Penetration enhancers. Adv. Drug Deliv. Rev..

[111] Cheung K., Han T., Das D.B. (2014). Effect of Force of Microneedle Insertion on the Permeability of Insulin in Skin. J. Diabetes Sci. Technol..

[112] Laurent A., Mistretta F., Bottigioli D., Dahel K., Goujon C., Nicolas J.F., Hennino A., Laurent P.E. (2007). Echographic measurement of skin thickness in adults by high frequency ultrasound to assess the appropriate microneedle length for intradermal delivery of vaccines. Vaccine.

[113] Hegde N.R., Kaveri S., Bayry J. (2011). Recent advances in the administration of vaccines for infectious diseases: Microneedles as painless delivery devices for mass vaccination. Drug Discov. Today.

[114] Chen S., Wu D., Liu Y., Huang Y., Xu H., Gao W., Zhang J., Sun J., Zhuang J. (2020). Optimal scaling analysis of polymeric microneedle length and its effect on transdermal insulin delivery. J. Drug Deliv. Sci. Technol..

[115] Li Q.Y., Zhang J.N., Chen B.Z., Wang Q.L., Guo X.D. (2017). A solid polymer microneedle patch pretreatment enhances the permeation of drug molecules into the skin. RSC Adv..

[116] Chen B., Liu J.L., Li Q.Y., Wang Z.N., Zhang X.P., Shen C.B., Cui Y., Guo X.D. (2019). Safety Evaluation of Solid Polymer Microneedles in Human Volunteers at Different Application Sites. ACS Appl. Bio Mater..

[117] Merad M., Ginhoux F., Collin M. (2008). Origin, homeostasis and function of Langerhans cells and other langerin-expressing dendritic cells. Nat. Rev. Immunol..

[118] Donnelly R.F., Singh R.R.T., Tunney M., Morrow D.I.J., McCarron P., O’Mahony C., Woolfson A.D. (2009). Microneedle Arrays Allow Lower Microbial Penetration Than Hypodermic Needles In Vitro. Pharm. Res..

[119] Coyne J., Davis B., Kauffman D., Zhao N., Wang Y. (2017). Polymer Microneedle Mediated Local Aptamer Delivery for Blocking the Function of Vascular Endothelial Growth Factor. ACS Biomater. Sci. Eng..

[120] Dul M., Stefanidou M., Porta P., Serve J., O’Mahony C., Malissen B., Henri S., Levin Y., Kochba E., Wong F.S. (2017). Hydrodynamic gene delivery in human skin using a hollow microneedle device. J. Control. Release.

[121] Jeong H.-R., Kim J.-Y., Kim S.-N., Park J.-H. (2018). Local dermal delivery of cyclosporin A, a hydrophobic and high molecular weight drug, using dissolving microneedles. Eur. J. Pharm. Biopharm..

[122] Gomaa Y.A., Garland M.J., McInnes F.J., Donnelly R.F., El-Khordagui L.K., Wilson C.G. (2012). Flux of ionic dyes across microneedle-treated skin: Effect of molecular characteristics. Int. J. Pharm..

[123] Davis S.P., Landis B.J., Adams Z.H., Allen M.G., Prausnitz M.R. (2004). Insertion of microneedles into skin: Measurement and prediction of insertion force and needle fracture force. J. Biomech..

[124] Olatunji O., Igwe C.C., Ahmed A.S., Alhassan D.O.A., Asieba G.O., Das Diganta B. (2014). Microneedles from fish scale biopolymer. J. Appl. Polym. Sci..

[125] Al-Qallaf B., Das D.B. (2008). Optimization of square microneedle arrays for increasing drug permeability in skin. Chem. Eng. Sci..

[126] Olatunji O., Das D.B., Nassehi V. (2012). Modelling Transdermal Drug Delivery Using Microneedles: Effect of Geometry on Drug Transport Behaviour. J. Pharm. Sci..

[127] Davidson A., Al-Qallaf B., Das D.B. (2008). Transdermal drug delivery by coated microneedles: Geometry effects on effective skin thickness and drug permeability. Chem. Eng. Res. Des..

[128] Al-Qallaf B., Das D.B. (2009). Optimizing Microneedle Arrays to Increase Skin Permeability for Transdermal Drug Delivery. Ann. N. Y. Acad. Sci..

[129] Flaten G.E., Palac Z., Engesland A., Filipović-Grčić J., Vanić Ž., Škalko-Basnet N. (2015). In vitro skin models as a tool in optimization of drug formulation. Eur. J. Pharm. Sci..

[130] Andrews S.N., Jeong E., Prausnitz M.R. (2012). Transdermal Delivery of Molecules is Limited by Full Epidermis, Not Just Stratum Corneum. Pharm. Res..

[131] Sandrakov G.V., Lyashko S.I., Bondar E.S., Lyashko N.I. (2019). Modeling and Optimization of Microneedle Systems. J. Autom. Inf. Sci..

[132] Loizidou E.Z., Williams N.A., Barrow D.A., Eaton M.J., McCrory J., Evans S., Allender C.J. (2015). Structural characterisation and transdermal delivery studies on sugar microneedles: Experimental and finite element modelling analyses. Eur. J. Pharm. Biopharm..

[133] Zhang N., Das D.B., Rielly C.D. (2015). Microneedle assisted micro-particle delivery by gene guns: Mathematical model formulation and experimental verification. Chem. Eng. Sci..

[134] Zhang N., Rielly C.D., Das D.B. (2014). Microneedle-assisted microparticle delivery by gene guns: Experiments and modeling on the effects of particle characteristics. Drug Deliv..

[135] Ronnander P., Simon L., Spilgies H., Koch A. (2018). Modelling the in-vitro dissolution and release of sumatriptan succinate from polyvinylpyrrolidone-based microneedles. Eur. J. Pharm. Sci..

[136] Zhang R., Zhang P., Dalton C., Jullien G.A. (2009). Modeling of drug delivery into tissues with a microneedle array using mixture theory. Biomech. Model. Mechanobiol..

[137] Kearney M.-C., Caffarel-Salvador E., Fallows S.J., McCarthy H.O., Donnelly R.F. (2016). Microneedle-mediated delivery of donepezil: Potential for improved treatment options in Alzheimer’s disease. Eur. J. Pharm. Biopharm..

[138] Tarbox T.N., Watts A.B., Cui Z., Williams R.O. (2017). An update on coating/manufacturing techniques of microneedles. Drug Deliv. Transl. Res..

[139] Li J., Liu B., Zhou Y., Chen Z., Jiang L., Yuan W., Liang L. (2017). Fabrication of a Ti porous microneedle array by metal injection molding for transdermal drug delivery. PLoS ONE.

[140] Lee K., Song H.B., Cho W., Kim J.H., Kim J.H., Ryu W. (2018). Intracorneal injection of a detachable hybrid microneedle for sustained drug delivery. Acta Biomater..

[141] Li J., Zhou Y., Yang J., Ye R., Gao J., Ren L., Liu B., Liang L., Jiang L. (2019). Fabrication of gradient porous microneedle array by modified hot embossing for transdermal drug delivery. Mater. Sci. Eng. C.

[142] Gholami S., Mohebi M.-M., Hajizadeh-Saffar E., Ghanian M.-H., Zarkesh I., Baharvand H. (2019). Fabrication of microporous inorganic microneedles by centrifugal casting method for transdermal extraction and delivery. Int. J. Pharm..

[143] Han M., Kim D.K., Kang S.H., Yoon H.-R., Kim B.-Y., Lee S.S., Kim K.D., Lee H.G. (2009). Improvement in antigen-delivery using fabrication of a grooves-embedded microneedle array. Sens. Actuators B Chem..

[144] Keum D.H., Jung H.S., Wang T., Shin M.H., Kim Y.-E., Kim K.H., Ahn G.-O., Hahn S.K. (2015). Cancer Detection: Microneedle Biosensor for Real-Time Electrical Detection of Nitric Oxide for In Situ Cancer Diagnosis During Endomicroscopy (Adv. Healthcare Mater. 8/2015). Adv. Healthc. Mater..

[145] Creighton R.L., Woodrow K.A. (2018). Microneedle-Mediated Vaccine Delivery to the Oral Mucosa. Adv. Healthc Mater..

[146] Rodgers A.M., Cordeiro A.S., Donnelly R.F. (2019). Technology update: Dissolvable microneedle patches for vaccine delivery. Med. Devices Évid. Res..

[147] Sullivan S.P., Murthy N., Prausnitz M.R. (2008). Minimally Invasive Protein Delivery with Rapidly Dissolving Polymer Microneedles. Adv. Mater..

[148] Lee K., Lee H.C., Lee D.-S., Jung H. (2010). Drawing Lithography: Three-Dimensional Fabrication of an Ultrahigh-Aspect-Ratio Microneedle. Adv. Mater..

[149] Vecchione R., Coppola S., Esposito E., Casale C., Vespini V., Grilli S., Ferraro P., Netti P.A. (2014). Electro-Drawn Drug-Loaded Biodegradable Polymer Microneedles as a Viable Route to Hypodermic Injection. Adv. Funct. Mater..

[150] Cao Y., Tao Y., Zhou Y., Gui S. (2016). Development of sinomenine hydrochloride-loaded polyvinylalcohol/maltose microneedle for transdermal delivery. J. Drug Deliv. Sci. Technol..

[151] Chandrasekharan A., Hwang Y.J., Seong K.-Y., Park S., Kim S., Yang S.Y. (2019). Acid-Treated Water-Soluble Chitosan Suitable for Microneedle-Assisted Intracutaneous Drug Delivery. Pharmaceutics.

[152] Sethi K., Bozin M., Jabane T., McMullin R., Cook D., Forsyth R., Dodds L., Putra L.J. (2017). Thermo-expandable prostatic stents for bladder outlet obstruction in the frail and elderly population: An underutilized procedure?. Investig. Clin. Urol..

[153] González-Vázquez P., Larrañeta E., McCrudden M.T., Jarrahian C., Rein-Weston A., Quintanar-Solares M., Zehrung D., McCarthy H., Courtenay A., Donnelly R.F. (2017). Transdermal delivery of gentamicin using dissolving microneedle arrays for potential treatment of neonatal sepsis. J. Control. Release.

[154] Li F., Song S., Guo Y., Zhao Q., Zhang X., Pan W., Yang X. (2013). Preparation and pharmacokinetics evaluation of oral self-emulsifying system for poorly water-soluble drug Lornoxicam. Drug Deliv..

[155] Yang J., Liu X., Fu Y., Song Y. (2019). Recent advances of microneedles for biomedical applications: Drug delivery and beyond. Acta Pharm. Sin. B.

[156] Chen Z., Ye R., Yang J., Lin Y., Lee W., Li J., Ren L., Liu B., Jiang L. (2019). Rapidly Fabricated Microneedle Arrays Using Magnetorheological Drawing Lithography for Transdermal Drug Delivery. ACS Biomater. Sci. Eng..

[157] Lee K., Jung H. (2012). Drawing lithography for microneedles: A review of fundamentals and biomedical applications. Biomaterials.

[158] Zylberberg C., Matosevic S. (2016). Pharmaceutical liposomal drug delivery: A review of new delivery systems and a look at the regulatory landscape. Drug Deliv..

[159] Pedde R.D., Mirani B., Navaei A., Styan T., Wong S., Mehrali M., Thakur A., Mohtaram N.K., Bayati A., Dolatshahi-Pirouz A. (2017). Emerging Biofabrication Strategies for Engineering Complex Tissue Constructs. Adv. Mater..

[160] Johnson A.R., Procopio A.T. (2019). Low cost additive manufacturing of microneedle masters. 3D Print. Med..

[161] Lim S.H., Ng J.Y., Kang L. (2017). Three-dimensional printing of a microneedle array on personalized curved surfaces for dual-pronged treatment of trigger finger. Biofabrication.

[162] Krieger K.J., Bertollo N., Dangol M., Sheridan J.T., Lowery M.M., O’Cearbhaill E.D. (2019). Simple and customizable method for fabrication of high-aspect ratio microneedle molds using low-cost 3D printing. Microsyst. Nanoeng..

[163] Mistilis M.J., Bommarius A.S., Prausnitz M.R. (2015). Development of a Thermostable Microneedle Patch for Influenza Vaccination. J. Pharm. Sci..

[164] Economidou S.N., Pere C.P.P., Reid A., Uddin J., Windmill J., Lamprou D.A., Douroumis D. (2019). 3D printed microneedle patches using stereolithography (SLA) for intradermal insulin delivery. Mater. Sci. Eng. C.

[165] Economidou S., Pere C.P., Okereke M., Douroumis D. (2021). Optimisation of Design and Manufacturing Parameters of 3D Printed Solid Microneedles for Improved Strength, Sharpness, and Drug Delivery. Micromachines.

[166] Economidou S.N., Uddin J., Marques M.J., Douroumis D., Sow W.T., Li H., Reid A., Windmill J.F., Podoleanu A. (2021). A novel 3D printed hollow microneedle microelectromechanical system for controlled, personalized transdermal drug delivery. Addit. Manuf..

[167] Economidou S.N., Lamprou D.A., Douroumis D. (2018). 3D printing applications for transdermal drug delivery. Int. J. Pharm..

[168] Pere C.P.P., Economidou S.N., Lall G., Ziraud C., Boateng J.S., Alexander B.D., Lamprou D., Douroumis D. (2018). 3D printed microneedles for insulin skin delivery. Int. J. Pharm..

[169] Ross S., Scoutaris N., Lamprou D., Mallinson D., Douroumis D. (2015). Inkjet printing of insulin microneedles for transdermal delivery. Drug Deliv. Transl. Res..

[170] Ovsianikov A., Chichkov B., Mente P., Monteiro-Riviere N., Doraiswamy A., Narayan R.J. (2007). Two Photon Polymerization of Polymer?Ceramic Hybrid Materials for Transdermal Drug Delivery. Int. J. Appl. Ceram. Technol..

[171] Yao W., Li D., Zhao Y., Zhan Z., Jin G., Liang H., Yang R. (2019). 3D Printed Multi-Functional Hydrogel Microneedles Based on High-Precision Digital Light Processing. Micromachines.

[172] Cordeiro A.S., Tekko I., Jomaa M.H., Vora L., McAlister E., Volpe-Zanutto F., Nethery M., Baine P.T., Mitchell N., McNeill D.W. (2020). Two-Photon Polymerisation 3D Printing of Microneedle Array Templates with Versatile Designs: Application in the Development of Polymeric Drug Delivery Systems. Pharm. Res..

[173] Farias C., Lyman R., Hemingway C., Chau H., Mahacek A., Bouzos E., Mobed-Miremadi M. (2018). Three-Dimensional (3D) Printed Microneedles for Microencapsulated Cell Extrusion. Bioengineering.

[174] Liu G., Kong Y., Wang Y., Luo Y., Fan X., Xie X., Yang B.-R., Wu M.X. (2020). Microneedles for transdermal diagnostics: Recent advances and new horizons. Biomaterials.

[175] Lee J.W., Han M.-R., Park J.-H. (2012). Polymer microneedles for transdermal drug delivery. J. Drug Target..

[176] Li F., Li X., He R., Cheng J., Ni Z., Zhao G. (2020). Preparation and evaluation of poly(D, L-lactic acid)/poly(l-lactide-*co*-ε-caprolactone) blends for tunable sirolimus release. Colloids Surfaces A Physicochem. Eng. Asp..

[177] Kim Y.C., Lee J.W., Esser E.S., Kalluri H., Joyce J.C., Compans R.W., Skountzou I., Prausnitz M.R. (2021). Fabrication of microneedle patches with lyophilized influenza vaccine suspended in organic solvent. Drug Deliv. Transl. Res..

[178] Liu J.L., Feng Y.H., Zhang X.P., Zhu D.D., Zhang L.Q., Guo X.D. (2020). Experimental and theoretical studies of drug-polymer interactions to control the drug distributions in dissolving microneedles. J. Ind. Eng. Chem..

[179] Long L.-Y., Zhang J., Yang Z., Guo Y., Hu X., Wang Y. (2020). Transdermal delivery of peptide and protein drugs: Strategies, advantages and disadvantages. J. Drug Deliv. Sci. Technol..

[180] Xiao Q., Guo T., Li J., Li L., Chen K., Zhou L., Wu W., So K.-F., Ramakrishna S., Liu B. (2021). Macrophage polarization induced by sustained release of 7,8-DHF from aligned PLLA fibers potentially for neural stem cell neurogenesis. Mater. Sci. Eng. C.

[181] Nguyen H.X., Bozorg B.D., Kim Y., Wieber A., Birk G., Lubda D., Banga A.K. (2018). Poly (vinyl alcohol) microneedles: Fabrication, characterization, and application for transdermal drug delivery of doxorubicin. Eur. J. Pharm. Biopharm..

[182] Battisti M., Vecchione R., Casale C., Pennacchio F.A., Lettera V., Jamaledin R., Profeta M., Di Natale C., Imparato G., Urciuolo F. (2019). Non-invasive Production of Multi-Compartmental Biodegradable Polymer Microneedles for Controlled Intradermal Drug Release of Labile Molecules. Front. Bioeng. Biotechnol..

[183] Arshad M.S., Fatima S., Nazari K., Ali R., Farhan M., Muhammad S.A., Abbas N., Hussain A., Kucuk I., Chang M.-W. (2019). Engineering and characterisation of BCG-loaded polymeric microneedles. J. Drug Target..

[184] Kolli C.S., Banga A.K. (2007). Characterization of Solid Maltose Microneedles and their Use for Transdermal Delivery. Pharm. Res..

[185] Shah V., Choudhury B.K. (2017). Fabrication, Physicochemical Characterization, and Performance Evaluation of Biodegradable Polymeric Microneedle Patch System for Enhanced Transcutaneous Flux of High Molecular Weight Therapeutics. AAPS PharmSciTech.

[186] Ogunjimi A., Fiegel J., Brogden N.K. (2020). Design and Characterization of Spray-Dried Chitosan-Naltrexone Microspheres for Microneedle-Assisted Transdermal Delivery. Pharmaceutics.

[187] Friedmann A., Cismak A., Tautorat C., Koester P.J., Baumann W., Held J., Gaspar J., Ruther P., Paul O., Heilmann A. (2011). FIB Preparation and SEM Investigations for Three-Dimensional Analysis of Cell Cultures on Microneedle Arrays. Scanning.

[188] Nejad H.R., Sadeqi A., Kiaee G., Sonkusale S. (2018). Low-cost and cleanroom-free fabrication of microneedles. Microsyst. Nanoeng..

[189] Ryan E., Garland M.J., Singh T.R.R., Bambury E., O’Dea J., Migalska K., Gorman S.P., McCarthy H., Gilmore B.F., Donnelly R.F. (2012). Microneedle-mediated transdermal bacteriophage delivery. Eur. J. Pharm. Sci..

[190] Pattarabhiran S.P., Saju A., Sonawane K.R., Manimaran R., Bhatnagar S., Roy G., Kulkarni R.B., Venuganti V.V.K. (2019). Dissolvable Microneedle-Mediated Transcutaneous Delivery of Tetanus Toxoid Elicits Effective Immune Response. AAPS PharmSciTech.

[191] Forouzandeh F., Borkholder D.A. (2020). Microtechnologies for inner ear drug delivery. Curr. Opin. Otolaryngol. Head Neck Surg..

[192] Migalska K., Morrow D.I.J., Garland M.J., Thakur R.S., Woolfson A.D., Donnelly R.F. (2011). Laser-Engineered Dissolving Microneedle Arrays for Transdermal Macromolecular Drug Delivery. Pharm. Res..

[193] Chen M., Quan G., Sun Y., Yang D., Pan X., Wu C. (2020). Nanoparticles-encapsulated polymeric microneedles for transdermal drug delivery. J. Control. Release.

[194] Upadhyay M., Vardhan H., Mishra B. (2020). Natural polymers composed mucoadhesive interpenetrating buoyant hydrogel beads of capecitabine: Development, characterization and in vivo scintigraphy. J. Drug Deliv. Sci. Technol..

[195] Chen Z., Han B., Liao L., Hu X., Hu Q., Gao Y., Qiu Y. (2020). Enhanced transdermal delivery of polydatin via a combination of inclusion complexes and dissolving microneedles for treatment of acute gout arthritis. J. Drug Deliv. Sci. Technol..

[196] Yu K., Yu X., Cao S., Wang Y., Zhai Y., Yang F., Yang X., Lu Y., Wu C., Xu Y. (2021). Layered dissolving microneedles as a need-based delivery system to simultaneously alleviate skin and joint lesions in psoriatic arthritis. Acta Pharm. Sin. B.

[197] Permana A.D., Mir M., Utomo E., Donnelly R.F. (2020). Bacterially sensitive nanoparticle-based dissolving microneedles of doxycycline for enhanced treatment of bacterial biofilm skin infection: A proof of concept study. Int. J. Pharm. X.

[198] Permana A.D., Paredes A., Volpe-Zanutto F., Anjani Q.K., Utomo E., Donnelly R.F. (2020). Dissolving microneedle-mediated dermal delivery of itraconazole nanocrystals for improved treatment of cutaneous candidiasis. Eur. J. Pharm. Biopharm..

[199] Tekko I.A., Chen G., Domínguez-Robles J., Thakur R.R.S., Hamdan I., Vora L., Larrañeta E., McElnay J.C., McCarthy H.O., Rooney M. (2020). Development and characterisation of novel poly (vinyl alcohol)/poly (vinyl pyrrolidone)-based hydrogel-forming microneedle arrays for enhanced and sustained transdermal delivery of methotrexate. Int. J. Pharm..

[200] Sabri A.H., Cater Z., Ogilvie J., Scurr D.J., Marlow M., Segal J. (2020). Characterisation of mechanical insertion of commercial microneedles. J. Drug Deliv. Sci. Technol..

[201] Hao Y., Chen Y., He X., Yang F., Han R., Yang C., Li W., Qian Z. (2020). Near-infrared responsive 5-fluorouracil and indocyanine green loaded MPEG-PCL nanoparticle integrated with dissolvable microneedle for skin cancer therapy. Bioact. Mater..

[202] Patel A., Cholkar K., Agrahari V., Mitra A.K. (2013). Ocular drug delivery systems: An overview. World J. Pharmacol..

[203] Oh J.-H., Park H.-H., Do K.-Y., Han M., Hyun D.-H., Kim C., Lee S.S., Hwang S.-J., Shin S.-C. (2008). Influence of the delivery systems using a microneedle array on the permeation of a hydrophilic molecule, calcein. Eur. J. Pharm. Biopharm..

[204] Zhao X., Zhang S., Yang G., Zhou Z., Gao Y. (2020). Exploring Trehalose on the Release of Levonorgestrel from Implantable PLGA Microneedles. Polymers.

[205] Ma Y., Gill H.S. (2014). Coating Solid Dispersions on Microneedles via a Molten Dip-Coating Method: Development and In Vitro Evaluation for Transdermal Delivery of a Water-Insoluble Drug. J. Pharm. Sci..

[206] Chen Y., Chen B.Z., Wang Q.L., Jin X., Guo X.D. (2017). Fabrication of coated polymer microneedles for transdermal drug delivery. J. Control. Release.

[207] Al Sulaiman D., Chang J.Y.H., Bennett N.R., Topouzi H., Higgins C.A., Irvine D.J., Ladame S. (2019). Hydrogel-Coated Microneedle Arrays for Minimally Invasive Sampling and Sensing of Specific Circulating Nucleic Acids from Skin Interstitial Fluid. ACS Nano.

[208] Bhatnagar S., Gadeela P.R., Thathireddy P., Venuganti V.V.K. (2019). Microneedle-based drug delivery: Materials of construction. J. Chem. Sci..

[209] Nguyen T.T., Choi J.-A., Kim J.S., Park H., Yang E., Lee W.J., Baek S.-K., Song M., Park J.-H. (2019). Skin immunization with third-generation hepatitis B surface antigen using microneedles. Vaccine.

[210] Naik S.P., Zade J.K., Sabale R.N., Pisal S.S., Menon R., Bankar S.G., Gairola S., Dhere R.M. (2017). Stability of heat stable, live attenuated Rotavirus vaccine (ROTASIIL^®^). Vaccine.

[211] Hwang C.S., Bremer P.T., Wenthur C.J., Ho S.O., Chiang S., Ellis B., Zhou B., Fujii G., Janda K.D. (2018). Enhancing Efficacy and Stability of an Antiheroin Vaccine: Examination of Antinociception, Opioid Binding Profile, and Lethality. Mol. Pharm..

[212] Clénet D. (2018). Accurate prediction of vaccine stability under real storage conditions and during temperature excursions. Eur. J. Pharm. Biopharm..

[213] Muruato A.E., Shan C., Fontes-Garfias C.R., Liu Y., Cao Z., Gao Q., Weaver S.C., Shi P.-Y. (2019). Genetic stability of live-attenuated Zika vaccine candidates. Antivir. Res..

[214] Bragazzi N.L., Orsi A., Ansaldi F., Gasparini R., Icardi G. (2016). Fluzone^®^ intra-dermal (Intanza^®^/Istivac^®^ Intra-dermal): An updated overview. Hum. Vaccines Immunother..

[215] Mistilis M.J., Joyce J., Esser E.S., Skountzou I., Compans R.W., Bommarius A.S., Prausnitz M.R. (2016). Long-term stability of influenza vaccine in a dissolving microneedle patch. Drug Deliv. Transl. Res..

[216] Kolluru C., Gomaa Y., Prausnitz M.R. (2018). Development of a thermostable microneedle patch for polio vaccination. Drug Deliv. Transl. Res..

[217] Demir Y.K., Akan Z., Kerimoglu O. (2013). Characterization of Polymeric Microneedle Arrays for Transdermal Drug Delivery. PLoS ONE.

[218] Li D., Hu D., Xu H., Patra H.K., Liu X., Zhou Z., Tang J., Slater N., Shen Y. (2021). Progress and perspective of microneedle system for anti-cancer drug delivery. Biomaterials.

[219] Courtenay A.J., McAlister E., McCrudden M.T., Vora L., Steiner L., Levin G., Levy-Nissenbaum E., Shterman N., Kearney M.-C., McCarthy H. (2020). Hydrogel-forming microneedle arrays as a therapeutic option for transdermal esketamine delivery. J. Control. Release.

[220] Mao J., Wang H., Xie Y., Fu Y., Li Y., Liu P., Du H., Zhu J., Dong L., Hussain M. (2019). Transdermal delivery of rapamycin with poor water-solubility by dissolving polymeric microneedles for anti-angiogenesis. J. Mater. Chem. B.

[221] Amer M., Chen R.K. (2020). Self-Adhesive Microneedles with Interlocking Features for Sustained Ocular Drug Delivery. Macromol. Biosci..

[222] Pawar S., Shende P. (2020). A comparative outlook on pharmacokinetics and antimalarial studies of artemether and lumefantrine-loaded microneedle patches and a dry suspension containing nanosponges. J. Drug Deliv. Sci. Technol..

[223] Yan Q., Wang W., Weng J., Zhang Z., Yin L., Yang Q., Guo F., Wang X., Chen F., Yang G. (2020). Dissolving microneedles for transdermal delivery of huperzine A for the treatment of Alzheimer’s disease. Drug Deliv..

[224] So J.-W., Park H.-H., Lee S.S., Kim D.-C., Shin S.-C., Cho C.-W. (2009). Effect of microneedle on the pharmacokinetics of ketoprofen from its transdermal formulations. Drug Deliv..

[225] Ling M.-H., Chen M.-C. (2013). Dissolving polymer microneedle patches for rapid and efficient transdermal delivery of insulin to diabetic rats. Acta Biomater..

[226] McCrudden M.T., Alkilani A.Z., McCrudden C.M., McAlister E., McCarthy H., Woolfson A.D., Donnelly R.F. (2014). Design and physicochemical characterisation of novel dissolving polymeric microneedle arrays for transdermal delivery of high dose, low molecular weight drugs. J. Control. Release.

[227] Xu Y., Li H., Fan L., Chen Y., Li L., Zhou X., Li R., Cheng Y., Chen H., Yuan Z. (2021). Development of photosensitizer-loaded lipid droplets for photothermal therapy based on thiophene analogs. J. Adv. Res..

[228] Balmert S.C., Carey C.D., Falo G.D., Sethi S.K., Erdos G., Korkmaz E., Falo L.D. (2020). Dissolving undercut microneedle arrays for multicomponent cutaneous vaccination. J. Control. Release.

[229] Hiew T.N., Tian Y.H., Tan H.M., Heng P.W.S. (2020). A mechanistic understanding of compression damage to the dissolubility of coated pellets in tablets. Eur. J. Pharm. Biopharm..

[230] Ahmad Z., Khan M.I., Siddique M.I., Sarwar H.S., Shahnaz G., Hussain S.Z., Bukhari N.I., Hussain I., Sohail M.F. (2020). Fabrication and Characterization of Thiolated Chitosan Microneedle Patch for Transdermal Delivery of Tacrolimus. AAPS PharmSciTech.

[231] Prausnitz M.R. (2017). Engineering Microneedle Patches for Vaccination and Drug Delivery to Skin. Annu. Rev. Chem. Biomol. Eng..

[232] Šimková K., Joost B., Imanidis G. (2020). Production of fast-dissolving low-density powders for improved lung deposition by spray drying of a nanosuspension. Eur. J. Pharm. Biopharm..

[233] Imono M., Uchiyama H., Yoshida S., Miyazaki S., Tamura N., Tsutsumimoto H., Kadota K., Tozuka Y. (2020). The elucidation of key factors for oral absorption enhancement of nanocrystal formulations: In vitro–in vivo correlation of nanocrystals. Eur. J. Pharm. Biopharm..

[234] Wangab D., Chengad D.B., Jia L., Niub L.J., Zhanga X.H., Conga Y., Caob R.H., Zhouc L., Baib F., Qiaoa Z.Y. (2021). Precise magnetic resonance imaging-guided sonodynamic therapy for drug-resistant bacterial deep infection. Biomaterials.

[235] Jeong H.-R., Bae J.-Y., Park J.-H., Baek S.-K., Kim G., Park M.-S., Park J.-H. (2020). Preclinical study of influenza bivalent vaccine delivered with a two compartmental microneedle array. J. Control. Release.

[236] Quinn H.L., Bonham L., Hughes C.M., Donnelly R. (2015). Design of a Dissolving Microneedle Platform for Transdermal Delivery of a Fixed-Dose Combination of Cardiovascular Drugs. J. Pharm. Sci..

[237] Zhang L., Hu K., Shao T., Hou L., Zhang S., Ye W., Josephson L., Meyer J.H., Zhang M.-R., Vasdev N. (2021). Recent developments on PET radiotracers for TSPO and their applications in neuroimaging. Acta Pharm. Sin. B.

[238] Lhernould M.S., Delchambre A. (2011). Innovative design of hollow polymeric microneedles for transdermal drug delivery. Microsyst. Technol..

[239] Hutton A.R., Quinn H.L., McCague P.J., Jarrahian C., Rein-Weston A., Coffey P.S., Gerth-Guyette E., Zehrung D., Larrañeta E., Donnelly R.F. (2018). Transdermal delivery of vitamin K using dissolving microneedles for the prevention of vitamin K deficiency bleeding. Int. J. Pharm..

[240] Li Y., Hu X., Dong Z., Chen Y., Zhao W., Wang Y., Zhang L., Chen M., Wu C., Wang Q. (2020). Dissolving Microneedle Arrays with Optimized Needle Geometry for Transcutaneous Immunization. Eur. J. Pharm. Sci..

[241] Yuzhakov V.V. (2010). Microneedle Array, Patch, and Applicator for Transdermal Drug Delivery. U.S. Patent.

[242] Cheng F., Carroll L., Joglekar M.V., Januszewski A.S., Wong K.K., Hardikar A., Jenkins A.J., Ma R.C.W. (2021). Diabetes, metabolic disease, and telomere length. Lancet Diabetes Endocrinol..

[243] Chen H., Zhu H., Zheng J., Mou D., Wan J., Zhang J., Shi T., Zhao Y., Xu H., Yang X. (2009). Iontophoresis-driven penetration of nanovesicles through microneedle-induced skin microchannels for enhancing transdermal delivery of insulin. J. Control. Release.

[244] Darvishha S., Amiri S. (2018). (Trans) dermal insulin delivery based on polymeric systems. Int. J. Polym. Mater..

[245] Vora L., Courtenay A., Tekko I., Larrañeta E., Donnelly R.F. (2020). Pullulan-based dissolving microneedle arrays for enhanced transdermal delivery of small and large biomolecules. Int. J. Biol. Macromol..

[246] Kim Y., Prausnitz M.R. (2021). Sensitive sensing of biomarkers in interstitial fluid. Nat. Biomed. Eng..

[247] Fontanillas P., Alipanahi B., Furlotte N.A., Johnson M., Wilson C.H., Pitts S.J., Gentleman R., Auton A. (2021). Disease risk scores for skin cancers. Nat. Commun..

[248] Salvio A.G., Requena M.B., Stringasci M.D., Bagnato V.S. (2021). Photodynamic therapy as a treatment option for multiple pigmented basal cell carcinoma: Long-term follow-up results. Photodiagnosis Photodyn. Ther..

[249] Dika E., Scarfì F., Ferracin M., Broseghini E., Marcelli E., Bortolani B., Campione E., Riefolo M., Ricci C., Lambertini M. (2020). Basal Cell Carcinoma: A Comprehensive Review. Int. J. Mol. Sci..

[250] de Albuquerque I.O., Nunes J., Longo J.P.F., Muehlmann L.A., Azevedo R.B. (2019). Photodynamic therapy in superficial basal cell carcinoma treatment. Photodiagnosis Photodyn. Ther..

[251] Fania L., Didona D., Morese R., Campana I., Coco V., Di Pietro F.R., Ricci F., Pallotta S., Candi E., Abeni D. (2020). Basal Cell Carcinoma: From Pathophysiology to Novel Therapeutic Approaches. Biomedicines.

[252] Cameron M.C., Lee E., Hibler B.P., Giordano C.N., Barker C.A., Mori S., Cordova M., Nehal K.S., Rossi A.M. (2019). Basal cell carcinoma. J. Am. Acad. Dermatol..

[253] Samarasinghe V., Madan V., Lear J.T. (2010). Focus on Basal Cell Carcinoma. J. Skin Cancer.

[254] Lanoue J., Goldenberg G. (2016). Basal Cell Carcinoma: A Comprehensive Review of Existing and Emerging Nonsurgical Therapies. J. Clin. Aesthet Dermatol..

[255] Lapteva M., Mignot M., Mondon K., Möller M., Gurny R., Kalia Y.N. (2019). Self-assembled mPEG-hexPLA polymeric nanocarriers for the targeted cutaneous delivery of imiquimod. Eur. J. Pharm. Biopharm..

[256] Naguib Y., Kumar A., Cui Z. (2014). The effect of microneedles on the skin permeability and antitumor activity of topical 5-fluorouracil. Acta Pharm. Sin. B.

[257] National AIDS Trust. https://www.nat.org.uk/about-hiv/hiv-statistics.

[258] Zaric M., Becker P.D., Hervouet C., Kalcheva P., Yus B.I., Cocita C., O’Neill L.A., Kwon S.-Y., Klavinskis L.S. (2017). Long-lived tissue resident HIV-1 specific memory CD8+ T cells are generated by skin immunization with live virus vectored microneedle arrays. J. Control. Release.

[259] Collins D.R., Gaiha G.D., Walker B.D. (2020). CD8+ T cells in HIV control, cure and prevention. Nat. Rev. Immunol..

[260] Chen Z., Julg B. (2020). Therapeutic Vaccines for the Treatment of HIV. Transl. Res..

[261] Zaric M., Lyubomska O., Touzelet O., Poux C., Al-Zahrani S., Fay F., Wallace L., Terhorst D., Malissen B., Henri S. (2013). Skin Dendritic Cell Targeting via Microneedle Arrays Laden with Antigen-Encapsulated Poly-d,l-lactide-*co*-Glycolide Nanoparticles Induces Efficient Antitumor and Antiviral Immune Responses. ACS Nano.

[262] Arya J.M., Dewitt K., Scott-Garrard M., Chiang Y.-W., Prausnitz M.R. (2016). Rabies vaccination in dogs using a dissolving microneedle patch. J. Control. Release.

[263] Mikszta J.A., Dekker J.P., Harvey N.G., Dean C.H., Brittingham J.M., Huang J., Sullivan V.J., Dyas B., Roy C., Ulrich R.G. (2006). Microneedle-Based Intradermal Delivery of the Anthrax Recombinant Protective Antigen Vaccine. Infect. Immun..

[264] Hiraishi Y., Nandakumar S., Choi S.-O., Lee J.W., Kim Y.-C., Posey J.E., Sable S.B., Prausnitz M.R. (2011). Bacillus Calmette-Guérin vaccination using a microneedle patch. Vaccine.

[265] Cafe R. (2011). How the Contraceptive Pill Changed Britain. BBC News.

[266] Mofidfar M., O’Farrell L., Prausnitz M.R. (2019). Pharmaceutical jewelry: Earring patch for transdermal delivery of contraceptive hormone. J. Control. Release.

[267] Sivasankaran S., Jonnalagadda S. (2021). Advances in controlled release hormonal technologies for contraception: A review of existing devices, underlying mechanisms, and future directions. J. Control. Release.

[268] Jang M., Baek S., Kang G., Yang H., Kim S., Jung H. (2020). Dissolving microneedle with high molecular weight hyaluronic acid to improve skin wrinkles, dermal density and elasticity. Int. J. Cosmet. Sci..

[269] Hou A., Cohen B., Haimovic A., Elbuluk N. (2017). Microneedling: A Comprehensive Review. Dermatol. Surg..

[270] Karimkhani C., Dellavalle R.P., Coffeng L.E., Flohr C., Hay R.J., Langan S., Nsoesie E.O., Ferrari A., Erskine H.E., Silverberg J.I. (2017). Global Skin Disease Morbidity and Mortality. JAMA Dermatol..

[271] Kang G., Tu T.N.T., Kim S., Yang H., Jang M., Jo D., Ryu J., Baek J., Jung H. (2018). Adenosine-loaded dissolving microneedle patches to improve skin wrinkles, dermal density, elasticity and hydration. Int. J. Cosmet. Sci..

[272] Rendon A., Schäkel K. (2019). Psoriasis Pathogenesis and Treatment. Int. J. Mol. Sci..

[273] Das A., Datta D., Kassir M., Wollina U., Galadari H., Lotti T., Jafferany M., Grabbe S., Goldust M. (2020). Acanthosis nigricans: A review. J. Cosmet. Dermatol..

[274] Armstrong A.W., Read C. (2020). Pathophysiology, Clinical Presentation, and Treatment of Psoriasis. JAMA.

[275] Sabri A.H., Ogilvie J., Abdulhamid K., Shpadaruk V., McKenna J., Segal J., Scurr D., Marlow M. (2019). Expanding the applications of microneedles in dermatology. Eur. J. Pharm. Biopharm..

[276] Korkmaz E., Friedrich E., Ramadan M.H., Erdos G., Mathers A.R., Ozdoganlar B., Washburn N.R., Falo L.D. (2015). Therapeutic intradermal delivery of tumor necrosis factor-alpha antibodies using tip-loaded dissolvable microneedle arrays. Acta Biomater..

[277] Konicke K., Olasz E. (2016). Successful Treatment of Recalcitrant Plantar Warts With Bleomycin and Microneedling. Dermatol. Surg..

[278] García-Oreja S., Álvaro-Afonso F.J., García-Álvarez Y., García-Morales E., Sanz-Corbalán I., Martínez J.L.L. (2020). Topical treatment for plantar warts: A systematic review. Dermatol. Ther..

[279] Ryu H.R., Jeong H.-R., Seon-Woo H.-S., Kim J.S., Lee S.K., Kim H.J., Baek J.O., Park J.-H., Roh J.Y. (2017). Efficacy of a bleomycin microneedle patch for the treatment of warts. Drug Deliv. Transl. Res..

[280] Kaul S., Caldito E.G., Jakhar D., Kaur I., Kwatra S.G., Mehta S. (2021). Comparative efficacy and safety of intralesional bleomycin relative to topical bleomycin with microneedling in the treatment of warts: A systematic review. J. Am. Acad. Dermatol..

[281] Zvezdin V., Kasatkina T., Kasatkin I., Gavrilova M., Kazakova O. (2020). Microneedle patch based on dissolving, detachable microneedle technology for improved skin quality of the periorbital region. Part 2: Clinical Evaluation. Int. J. Cosmet. Sci..

[282] Lee A.-R.C. (2019). Microneedle-mediated delivery of cosmeceutically relevant nucleoside and peptides in human skin: Challenges and strategies for dermal delivery. J. Pharm. Investig..

[283] Bhatnagar S., Kumari P., Pattarabhiran S.P., Venuganti V.V.K. (2018). Zein Microneedles for Localized Delivery of Chemotherapeutic Agents to Treat Breast Cancer: Drug Loading, Release Behavior, and Skin Permeation Studies. AAPS PharmSciTech.

[284] An J.H., Lee H.J., Yoon M.S., Kim D.H. (2019). Anti-Wrinkle Efficacy of Cross-Linked Hyaluronic Acid-Based Microneedle Patch with Acetyl Hexapeptide-8 and Epidermal Growth Factor on Korean Skin. Ann. Dermatol..

[285] He M., Yang G., Zhang S., Zhao X., Gao Y. (2018). Dissolving Microneedles Loaded With Etonogestrel Microcrystal Particles for Intradermal Sustained Delivery. J. Pharm. Sci..

[286] Lee H.S., Lee H.S., Lee H.S., Ryu H.R., Ryu H.R., Ryu H.R., Roh J.Y., Roh J.Y., Roh J.Y., Park J.-H. (2016). Bleomycin-Coated Microneedles for Treatment of Warts. Pharm. Res..

[287] Courtenay A.J., McCrudden M.T.C., McAvoy K.J., McCarthy H.O., Donnelly R.F. (2018). Microneedle-Mediated Transdermal Delivery of Bevacizumab. Mol. Pharm..

[288] Matsuo K., Okamoto H., Kawai Y., Quan Y.-S., Kamiyama F., Hirobe S., Okada N., Nakagawa S. (2014). Vaccine efficacy of transcutaneous immunization with amyloid β using a dissolving microneedle array in a mouse model of Alzheimer’s disease. J. Neuroimmunol..

[289] Luo Z., Sun W., Fang J., Lee K., Li S., Gu Z., Dokmeci M.R., Khademhosseini A. (2018). Biodegradable Gelatin Methacryloyl Microneedles for Transdermal Drug Delivery. Adv. Healthc. Mater..

[290] Dillon C., Hughes H., O’Reilly N., Allender C.J., Barrow D.A., McLoughlin P. (2019). Dissolving microneedle based transdermal delivery of therapeutic peptide analogues. Int. J. Pharm..

[291] Tas C., Joyce J., Nguyen H.X., Eangoor P., Knaack J.S., Banga A.K., Prausnitz M.R. (2017). Dihydroergotamine mesylate-loaded dissolving microneedle patch made of polyvinylpyrrolidone for management of acute migraine therapy. J. Control. Release.

[292] Courtenay A.J., Rodgers A.M., McCrudden M.T.C., McCarthy H.O., Donnelly R.F. (2018). Novel Hydrogel-Forming Microneedle Array for Intradermal Vaccination in Mice Using Ovalbumin as a Model Protein Antigen. Mol. Pharm..

[293] Nalluri B.N., Uppuluri C., Devineni J., Nayak A., Nair K.J., Whiteside B.R., Das D.B. (2017). Effect of microneedles on transdermal permeation enhancement of amlodipine. Drug Deliv. Transl. Res..

[294] Machekposhti S.A., Soltani M., Najafizadeh P., Ebrahimi S., Chen P. (2017). Biocompatible polymer microneedle for topical/dermal delivery of tranexamic acid. J. Control. Release.

[295] Al-Japairai K.A.S., Mahmood S., Almurisi S.H., Venugopal J.R., Hilles A.R., Azmana M., Raman S. (2020). Current trends in polymer microneedle for transdermal drug delivery. Int. J. Pharm..

[296] Tucak A., Sirbubalo M., Hindija L., Rahić O., Hadžiabdić J., Muhamedagić K., Čekić A., Vranić E. (2020). Microneedles: Characteristics, Materials, Production Methods and Commercial Development. Micromachines.

[297] Ingrole R.S., Azizoglu E., Dul M., Birchall J.C., Gill H.S., Prausnitz M.R. (2021). Trends of microneedle technology in the scientific literature, patents, clinical trials and internet activity. Biomaterials.

[298] Ito S., Hirobe S., Kawakita T., Saito M., Quan Y.-S., Kamiyama F., Ishii K.J., Nagao M., Fujisawa T., Tachibana M. (2020). Characteristic of K3 (CpG-ODN) as a Transcutaneous Vaccine Formulation Adjuvant. Pharmacology.

[299] Bhattacharjee S., Patanwala A.E., Lo-Ciganic W., Malone D.C., Lee J.K., Knapp S.M., Warholak T., Burke W.J. (2019). Alzheimer’s disease medication and risk of all-cause mortality and all-cause hospitalization: A retrospective cohort study. Alzheimer’s Dementia Transl. Res. Clin. Interv..

[300] Lim J., Tahk D., Yu J., Min D.-H., Jeon N.L. (2018). Design rules for a tunable merged-tip microneedle. Microsyst. Nanoeng..

[301] Larrañeta E., McCrudden M.T.C., Courtenay A., Donnelly R.F. (2016). Microneedles: A New Frontier in Nanomedicine Delivery. Pharm. Res..

[302] Micronjet^®^. https://www.nanopass.com/product/.

[303] Levin Y., Kochba E., Hung I., Kenney R. (2015). Intradermal vaccination using the novel microneedle device MicronJet600: Past, present, and future. Hum. Vaccines Immunother..

[304] Microstructured Transdermal Systems (MTS). https://kindevadd.com/technologies/#mts.

[305] Li X., Xu Q., Zhang P., Zhao X., Wang Y. (2019). Cutaneous microenvironment responsive microneedle patch for rapid gene release to treat subdermal tumor. J. Control. Release.

[306] Nayak A., Babla H., Han T., Das D.B. (2014). Lidocaine carboxymethylcellulose with gelatine co-polymer hydrogel delivery by combined microneedle and ultrasound. Drug Deliv..

[307] BD Technologies. https://drugdeliverysystems.bd.com/products-and-services/products/self-injection-systems..

[308] Ma G., Wu C. (2017). Microneedle, bio-microneedle and bio-inspired microneedle: A review. J. Control. Release.

[309] Lim D.-J., Vines J.B., Park H., Lee S.-H. (2018). Microneedles: A versatile strategy for transdermal delivery of biological molecules. Int. J. Biol. Macromol..

[310] Yadav V., Sharma P.K., Murty U.S., Mohan N.H., Thomas R., Dwivedy S.K., Banerjee S. (2021). 3D Printed Hollow Microneedles Array using Stereolithography for Efficient Transdermal Delivery of Rifampicin. Int. J. Pharm..

[311] Vescovo P., Rettby N., Ramaniraka N., Liberman J., Hart K., Cachemaille A., Piveteau L.-D., Zanoni R., Bart P.-A., Pantaleo G. (2017). Safety, tolerability and efficacy of intradermal rabies immunization with DebioJect™. Vaccine.

[312] Gualeni B., Coulman S., Shah D., Eng P., Ashraf H., Vescovo P., Blayney G., Piveteau L.-D., Guy O., Birchall J. (2017). Minimally invasive and targeted therapeutic cell delivery to the skin using microneedle devices. Br. J. Dermatol..

[313] Prymula R., Usluer G., Altinel S., Sichova R., Weber F. (2012). Acceptance and Opinions of Intanza/IDflu Intradermal Influenza Vaccine in the Czech Republic and Turkey. Adv. Ther..

[314] Han S.H., Woo J.H., Weber F., Kim W.J., Peck K.R., Kim S.I., Choi Y.H., Kim J.M. (2013). Immunogenicity and safety of Intanza^®^/IDflu^®^ intradermal influenza vaccine in South Korean adults: A multicenter, randomized trial. Hum. Vaccines Immunother..

[315] FDA (2020). Fluzone, Fluzone High-Dose and Fluzone Intradermal.

[316] Nguyen H.X., Banga A.K. (2018). Delivery of Methotrexate and Characterization of Skin Treated by Fabricated PLGA Microneedles and Fractional Ablative Laser. Pharm. Res..

[317] Jeong H.-R., Jun H., Cha H.-R., Lee J.M., Park J.-H. (2020). Safe Coated Microneedles with Reduced Puncture Occurrence after Administration. Micromachines.

[318] QtryptaTM. https://ir.zosanopharma.com/news-releases/news-release-details/zosano-pharma-receives-complete-response-letter-fda-qtryptatm.

[319] MTS Roller^TM^. https://www.clinicalresolution.com/main/whatismts.html.

[320] MTSRoller. https://www.mtsroller.com/.

[321] FDA (2020). Microneedling Devices.

[322] Micro-TransTM. https://www.fiercebiotech.com/biotech/valeritas-receives-fda-510-k-clearance-for-v-gotm-disposable-insulin-delivery-device-for.

[323] H-PatchTM Technology. https://www.globenewswire.com/news-release/2019/10/29/1936883/0/en/Valeritas-Presents-Positive-h-Patch-Apomorphine-Study-Data-at-the-World-Congress-of-Neurology-WCN-2019.html.

[324] Matriano J.A., Cormier M., Johnson J., Young W.A., Buttery M., Nyam K., Daddona P.E. (2002). Macroflux^®^ Microprojection Array Patch Technology: A New and Efficient Approach for Intracutaneous Immunization. Pharm. Res..

[325] Rodan + Fields. https://www.rodanandfields.com/pages/amp-md-roller-intensive-renewing-serum-lp.

[326] Indermun S., Luttge R., Choonara Y., Kumar P., du Toit L., Modi G., Pillay V. (2014). Current advances in the fabrication of microneedles for transdermal delivery. J. Control. Release.

[327] Cheung K., Das D.B. (2014). Microneedles for drug delivery: Trends and progress. Drug Deliv..

[328] Jung-Hwan P., Han M.-R., Kang N.-G., Park J.-H. (2015). Use of hollow microneedles for targeted delivery of phenylephrine to treat fecal incontinence. J. Control. Release.

[329] Xu Z., Rivera-Hernandez T., Chatterjee O., Walker M.J., Moyle P.M. (2020). Semisynthetic, self-adjuvanting vaccine development: Efficient, site-specific sortase A-mediated conjugation of Toll-like receptor 2 ligand FSL-1 to recombinant protein antigens under native conditions and application to a model group A streptococcal vaccine. J. Control. Release.

[330] Bonfante G., Lee H., Bao L., Park J., Takama N., Kim B. (2020). Comparison of polymers to enhance mechanical properties of microneedles for bio-medical applications. Micro Nano Syst. Lett..

[331] Wu D., Quan Y.-S., Kamiyama F., Kusamori K., Katsumi H., Sakane T., Yamamoto A. (2015). Improvement of Transdermal Delivery of Sumatriptan Succinate Using a Novel Self-dissolving Microneedle Array Fabricated from Sodium Hyaluronate in Rats. Biol. Pharm. Bull..

[332] Katsumi H., Tanaka Y., Hitomi K., Liu S., Quan Y.-S., Kamiyama F., Sakane T., Yamamoto A. (2017). Efficient Transdermal Delivery of Alendronate, a Nitrogen-Containing Bisphosphonate, Using Tip-Loaded Self-Dissolving Microneedle Arrays for the Treatment of Osteoporosis. Pharmaceutics.

[333] Felner E. (2013). Insulin Delivery Using Microneedles in Type 1 Diabetes. clinicaltrials.gov.

[334] University of British Columbia (2015). Controlled Comparison in Canadian Seniors of Seasonal Influenza Vaccines for 2011–2012. clinicaltrials.gov.

[335] University of California, Davis (2017). The Use of Microneedles in Photodynamic Therapy. clinicaltrials.gov.

[336] Radius Health, Inc. (2020). A Randomized, Double-Blind, Placebo-Controlled, Phase 2 Study of BA058 Administered Via a Coated Transdermal Microarray Delivery System (BA058 Transdermal) in Healthy Postmenopausal Women With Osteoporosis. clinicaltrials.gov.

[337] University of California, Davis (2017). The Use of Microneedles With Topical Botulinum Toxin for Treatment of Palmar Hyperhidrosis. clinicaltrials.gov.

[338] Prausnitz M. (2019). A Phase I Study of the Safety, Reactogenicity, Acceptability and Immunogenicity of Inactivated Influenza Vaccine Delivered by Microneedle Patch or by Hypodermic Needle. clinicaltrials.gov.

[339] Zosano Pharma Corporation (2018). Randomized, Double-Blind, Multi-Center, Parallel-Group, Dose-Ranging Comparison of the Safety and Efficacy of the ZP-Zolmitriptan Intracutaneous Microneedle Systems to Placebo for the Acute Treatment of Migraine. clinicaltrials.gov.

[340] Zosano Pharma Corporation (2020). A Long-Term, Open-Label Study to Evaluate the Safety of M207 (Zolmitriptan Intracutaneous Microneedle System) in the Acute Treatment of Migraine. clinicaltrials.gov.

[341] Yonsei University (2020). A Study on the Effectiveness and Safety Evaluation of Combination Therapy with 1927 nm Thulium Laser and Fractional Microneedle Radiofrequency Equipment for Improvement of Skin Aging. clinicaltrials.gov.

[342] Pulse Biosciences, Inc. (2020). Prospective, Open Label, Multi-Center, Non-Significant Risk Study of Nano-Pulse StimulationTM (NPSTM) Technology in Healthy Adults with Sebaceous Hyperplasia. clinicaltrials.gov.

[343] Pulse Biosciences, Inc. (2021). Prospective, Open-Label, Multi-Center, Non-Significant Risk Study of Nano-Pulse StimulationTM (NPSTM) Technology in Healthy Adults with Seborrheic Keratosis. clinicaltrials.gov.

[344] Falo J., Erdos G. (2020). Bioactive Components Conjugated to Dissolvable Substrates of Microneedle Arrays. U.S. Patent.

[345] Kato H. (2020). Microneedle and Method for Manufacturing Microneedle. U.S. Patent.

[346] Kaspar R.L., Speaker T. (2019). Microneedle Arrays Formed from Polymer Films. U.S. Patent.

[347] Jin T. (2019). CN Fabrication Process of Phase-Transition Microneedle Patch. U.S. Patent.

[348] Ghartey-Tagoe E., Wendorf J., Williams S., Singh P., Worsham R.W., Trautman J.C., Bayramov D., Bowers D.L., Klemm A.R., Klemm S.R. (2016). Method of Vaccine Delivery via Microneedle Arrays. U.S. Patent.

[349] Park J.-H., Prausnitz M.R. (2016). Microneedle Devices and Production Thereof. U.S. Patent.

[350] Falo J., Erdos G., Ozdoganlar O.B. (2014). Dissolvable Microneedle Arrays for Transdermal Delivery to Human Skin. U.S. Patent.

[351] Allen M.G., Prausnitz M.R., McAllister D.V., Cros F.P.M. (2014). Microneedle Devices and Methods of Manufacture and Use Thereof. U.S. Patent.

[352] Kaspar R.L., Speaker T. (2013). Microneedle Arrays Formed from Polymer Films. U.S. Patent.

[353] Chiou J.-C., Hung C.-C., Chang C.-W. (2008). Method for Fabricating Microneedle Array and Method for Fabricating Embossing Mold of Microneedle Array. U.S. Patent.

[354] Uppu D.S., Turvey M.E., Sharif A.R.M., Bidet K., He Y., Ho V., Tambe A.D., Lescar J., Tan E.Y., Fink K. (2020). Temporal release of a three-component protein subunit vaccine from polymer multilayers. J. Control. Release.

[355] Chang H., Zheng M., Chew S.W.T., Xu C. (2020). Advances in the Formulations of Microneedles for Manifold Biomedical Applications. Adv. Mater. Technol..

[356] Kim K.B., Lee W.-C., Cho C.-H., Park D.-S., Cho S.J., Shim Y.-B. (2019). Continuous glucose monitoring using a microneedle array sensor coupled with a wireless signal transmitter. Sens. Actuators B Chem..

[357] Pineda-Álvarez R.A., Bernad-Bernad M.J., Rodríguez-Cruz I.M., Escobar-Chávez J.J. (2020). Development and Characterization of Starch/Gelatin Microneedle Arrays Loaded with Lecithin–Gelatin Nanoparticles of Losartan for Transdermal Delivery. J. Pharm. Innov..

[358] Aksit A., Rastogi S., Nadal M.L., Parker A.M., Lalwani A.K., West A.C., Kysar J.W. (2020). Drug delivery device for the inner ear: Ultra-sharp fully metallic microneedles. Drug Deliv. Transl. Res..

[359] Chen Z., He J., Qi J., Zhu Q., Wu W., Lu Y. (2020). Long-acting microneedles: A progress report of the state-of-the-art techniques. Drug Discov. Today.

[360] Lee K., Goudie M.J., Tebon P., Sun W., Luo Z., Lee J., Zhang S., Fetah K., Kim H.-J., Xue Y. (2020). Non-transdermal microneedles for advanced drug delivery. Adv. Drug Deliv. Rev..

[361] Zhuang J., Rao F., Wu D., Huang Y., Xu H., Gao W., Zhang J., Sun J. (2020). Study on the fabrication and characterization of tip-loaded dissolving microneedles for transdermal drug delivery. Eur. J. Pharm. Biopharm..

[362] Pires L.R., Amado I.R., Gaspar J. (2020). Dissolving microneedles for the delivery of peptides—Towards tolerance-inducing vaccines. Int. J. Pharm..

[363] Ramalheiro A., Paris J.L., Silva B.F., Pires L.R. (2020). Rapidly dissolving microneedles for the delivery of cubosome-like liquid crystalline nanoparticles with sustained release of rapamycin. Int. J. Pharm..

[364] Azmana M., Mahmood S., Hilles A.R., Mandal U.K., Al-Japairai K.A.S., Raman S. (2020). Transdermal drug delivery system through polymeric microneedle: A recent update. J. Drug Deliv. Sci. Technol..

[365] Radhika C., Gnanavel B. (2021). Finite element analysis of polymer microneedle for transdermal drug delivery. Mater. Today Proc..

[366] Xie Y., Hillmyer M.A. (2020). Nanostructured Polymer Monoliths for Biomedical Delivery Applications. ACS Appl. Bio Mater..

[367] Roy G., Galigama R.D., Thorat V.S., Garg P., Venuganti V.V.K. (2020). Microneedle ocular patch: Fabrication, characterization, and ex-vivo evaluation using pilocarpine as model drug. Drug Dev. Ind. Pharm..

[368] Amodwala S., Kumar P., Thakkar H.P. (2017). Statistically optimized fast dissolving microneedle transdermal patch of meloxicam: A patient friendly approach to manage arthritis. Eur. J. Pharm. Sci..

[369] Larrañeta E., Moore J., Vicente-Pérez E.M., González-Vázquez P., Lutton R., Woolfson A.D., Donnelly R.F. (2014). A proposed model membrane and test method for microneedle insertion studies. Int. J. Pharm..

[370] Arai M., Kudo Y., Miki N. (2016). Polymer-based candle-shaped microneedle electrodes for electroencephalography on hairy skin. Jpn. J. Appl. Phys..

[371] Dardano P., Caliò A., Politi J., Rea I., Rendina I., De Stefano L. (2016). Optically monitored drug delivery patch based on porous silicon and polymer microneedles. Biomed. Opt. Express.

[372] Hong Y.-T., Bregante D.T., Lee J.C.-W., Seo Y., Kim D.-H., Lee Y.J., Schook L.B., Jeon H., Sung H.-J., Flaherty D.W. (2020). Catalytic microgelators for decoupled control of gelation rate and rigidity of the biological gels. J. Control. Release.

[373] Gao Y., Hou M., Yang R., Zhang L., Xu Z., Kang Y., Xue P. (2018). PEGDA/PVP Microneedles with Tailorable Matrix Constitutions for Controllable Transdermal Drug Delivery. Macromol. Mater. Eng..

[374] Chen Z., Ren L., Li J., Yao L., Chen Y., Liu B., Jiang L. (2018). Rapid fabrication of microneedles using magnetorheological drawing lithography. Acta Biomater..

[375] Puca E., De Luca R., Seehusen F., Rodriguez J.M.M., Neri D. (2020). Comparative evaluation of bolus and fractionated administration modalities for two antibody-cytokine fusions in immunocompetent tumor-bearing mice. J. Control. Release.

[376] Cheng H., Liu M., Du X., Xu J., Zhai Y., Ji J., He S., Zhai G. (2019). Recent progress of micro-needle formulations: Fabrication strategies and delivery applications. J. Drug Deliv. Sci. Technol..

[377] Zhang Y., Chai D., Gao M., Xu B., Jiang G. (2018). Thermal ablation of separable microneedles for transdermal delivery of metformin on diabetic rats. Int. J. Polym. Mater..

[378] Mandal A., Boopathy A.V., Lam L.K.W., Moynihan K.D., Welch M.E., Bennett N.R., Turvey M.E., Thai N., Van J.H., Love J.C. (2018). Cell and fluid sampling microneedle patches for monitoring skin-resident immunity. Sci. Transl. Med..

[379] Kim M., An J., Kim K.S., Choi M., Humar M., Kwok S.J., Dai T., Yun S.H. (2016). Optical lens-microneedle array for percutaneous light delivery. Biomed. Opt. Express.

[380] Manoukian O.S., Baker J.T., Rudraiah S., Arul M.R., Vella A.T., Domb A.J., Kumbar S.G. (2020). Functional polymeric nerve guidance conduits and drug delivery strategies for peripheral nerve repair and regeneration. J. Control. Release.

[381] Jayaneththi V., Aw K., Sharma M., Wen J., Svirskis D., McDaid A. (2019). Controlled transdermal drug delivery using a wireless magnetic microneedle patch: Preclinical device development. Sens. Actuators B Chem..

[382] Schossleitner K., O’Mahony C., Brandstätter S., Haslinger M.J., DeMuth S., Fechtig D., Petzelbauer P. (2018). Differences in biocompatibility of microneedles from cyclic olefin polymers with human endothelial and epithelial skin cells. J. Biomed. Mater. Res. Part A.

[383] Rajabi M., Roxhed N., Shafagh R.Z., Haraldson T., Fischer A.C., van der Wijngaart W., Stemme G., Niklaus F. (2016). Flexible and Stretchable Microneedle Patches with Integrated Rigid Stainless Steel Microneedles for Transdermal Biointerfacing. PLoS ONE.

[384] Kang N.-W., Kim S., Lee J.-Y., Kim K.-T., Choi Y., Oh Y., Kim J., Kim D.-D., Park J.-H. (2021). Microneedles for drug delivery: Recent advances in materials and geometry for preclinical and clinical studies. Expert Opin. Drug Deliv..

[385] Kathuria H., Lim D., Cai J., Chung B.G., Kang L. (2020). Microneedles with Tunable Dissolution Rate. ACS Biomater. Sci. Eng..

[386] Kim N.W., Kim S.-Y., Lee J.E., Yin Y., Lee J.H., Lim S.Y., Kim E.S., Duong H.T.T., Kim H.K., Kim S. (2018). Enhanced Cancer Vaccination by In Situ Nanomicelle-Generating Dissolving Microneedles. ACS Nano.

[387] Korkmaz E., Friedrich E.E., Ramadan M.H., Erdos G., Mathers A.R., Ozdoganlar O.B., Washburn N.R., Falo L.D. (2016). Tip-Loaded Dissolvable Microneedle Arrays Effectively Deliver Polymer-Conjugated Antibody Inhibitors of Tumor-Necrosis-Factor-Alpha Into Human Skin. J. Pharm. Sci..

[388] Zhu D.D., Wang Q.L., Liu X.B., Guo X.D. (2016). Rapidly separating microneedles for transdermal drug delivery. Acta Biomater..

[389] Zhu M., Liu Y., Jiang F., Cao J., Kundu S.C., Lu S. (2020). Combined Silk Fibroin Microneedles for Insulin Delivery. ACS Biomater. Sci. Eng..

[390] Zhang X., Si Z., Wang Y., Li Y., Xu C., Tian H. (2021). Polymerization and coordination synergistically constructed photothermal agents for macrophages-mediated tumor targeting diagnosis and therapy. Biomaterials.

[391] Quan P., Wan X., Tian Q., Liu C., Fang L. (2020). Dicarboxylic acid as a linker to improve the content of amorphous drug in drug-in-polymer film: Effects of molecular mobility, electrical conductivity and intermolecular interactions. J. Control. Release.

[392] Lee S., Lahiji S.F., Jang J., Jang M., Jung H. (2019). Micro-Pillar Integrated Dissolving Microneedles for Enhanced Transdermal Drug Delivery. Pharmaceutics.

[393] Yang P., Lu C., Qin W., Chen M., Quan G., Liu H., Wang L., Bai X., Pan X., Wu C. (2020). Construction of a core-shell microneedle system to achieve targeted co-delivery of checkpoint inhibitors for melanoma immunotherapy. Acta Biomater..

[394] Zeng Q., Gammon J.M., Tostanoski L., Chiu Y.-C., Jewell C.M. (2016). In Vivo Expansion of Melanoma-Specific T Cells Using Microneedle Arrays Coated with Immune-Polyelectrolyte Multilayers. ACS Biomater. Sci. Eng..

[395] Kim M.J., Park S.C., Rizal B., Guanes G., Baek S.-K., Park J.-H., Betz A.R., Choi S.-O. (2018). Fabrication of Circular Obelisk-Type Multilayer Microneedles Using Micro-Milling and Spray Deposition. Front. Bioeng. Biotechnol..

[396] Goud K.Y., Moonla C., Mishra R.K., Yu C., Narayan R., Litvan I., Wang J. (2019). Wearable Electrochemical Microneedle Sensor for Continuous Monitoring of Levodopa: Toward Parkinson Management. ACS Sens..

[397] Mishra R.K., Goud K.Y., Li Z.H., Moonla C., Mohamed M.A., Tehrani F., Teymourian H., Wang J. (2020). Continuous Opioid Monitoring along with Nerve Agents on a Wearable Microneedle Sensor Array. J. Am. Chem. Soc..

[398] Mohan A.V., Windmiller J.R., Mishra R.K., Wang J. (2017). Continuous minimally-invasive alcohol monitoring using microneedle sensor arrays. Biosens. Bioelectron..

[399] Sharma S., Saeed A., Johnson C., Gadegaard N., Cass A.E. (2017). Rapid, low cost prototyping of transdermal devices for personal healthcare monitoring. Sens. Bio-Sens. Res..

[400] Tang J., Wang J., Huang K., Ye Y., Su T., Qiao L., Hensley M.T., Caranasos T.G., Zhang J., Gu Z. (2018). Cardiac cell–integrated microneedle patch for treating myocardial infarction. Sci. Adv..

[401] Wang C., Ye Y., Hochu G.M., Sadeghifar H., Gu Z. (2016). Enhanced Cancer Immunotherapy by Microneedle Patch-Assisted Delivery of Anti-PD1 Antibody. Nano Lett..

[402] Dharadhar S., Majumdar A., Dhoble S., Patravale V. (2018). Microneedles for transdermal drug delivery: A systematic review. Drug Dev. Ind. Pharm..

[403] Ren L., Jiang Q., Chen K., Chen Z., Pan C., Jiang L. (2016). Fabrication of a Micro-Needle Array Electrode by Thermal Drawing for Bio-Signals Monitoring. Sensors.

[404] Abdelghany S., Tekko I.A., Vora L., Larrañeta E., Permana A.D., Donnelly R.F. (2019). Nanosuspension-Based Dissolving Microneedle Arrays for Intradermal Delivery of Curcumin. Pharmaceutics.

[405] Yu W., Jiang G., Liu D., Li L., Chen H., Liu Y., Huang Q., Tong Z., Yao J., Kong X. (2017). Fabrication of biodegradable composite microneedles based on calcium sulfate and gelatin for transdermal delivery of insulin. Mater. Sci. Eng. C.

[406] Tu K.T., Chung C.K. (2016). Rapid prototyping of biodegradable microneedle arrays by integrating CO2laser processing and polymer molding. J. Micromech.Microeng..

[407] Economidou S.N., Douroumis D. (2021). 3D printing as a transformative tool for microneedle systems: Recent advances, manufacturing considerations and market potential. Adv. Drug Deliv. Rev..

[408] Zhang X., Wang F., Yu Y., Chen G., Shang L., Sun L., Zhao Y. (2019). Bio-inspired clamping microneedle arrays from flexible ferrofluid-configured moldings. Sci. Bull..

[409] Teymourian H., Parrilla M., Sempionatto J.R., Montiel N.F., Barfidokht A., Van Echelpoel R., De Wael K., Wang J. (2020). Wearable Electrochemical Sensors for the Monitoring and Screening of Drugs. ACS Sens..

